# A Twenty-First Century Cancer Epidemic Caused by Obesity: The Involvement of Insulin, Diabetes, and Insulin-Like Growth Factors

**DOI:** 10.1155/2013/632461

**Published:** 2013-07-31

**Authors:** Rosalyne L. Westley, Felicity E. B. May

**Affiliations:** Northern Institute for Cancer Research, Faculty of Medical Sciences, University of Newcastle upon Tyne, Framlington Place, Newcastle upon Tyne NE2 4HH, UK

## Abstract

Obesity has reached epidemic proportions in the developed world. The progression from obesity to diabetes mellitus type 2, via metabolic syndrome, is recognised, and the significant associated increase in the risk of major human cancers acknowledged. We review the molecular basis of the involvement of morbidly high concentrations of endogenous or therapeutic insulin and of insulin-like growth factors in the progression from obesity to diabetes and finally to cancer. Epidemiological and biochemical studies establish the role of insulin and hyperinsulinaemia in cancer risk and progression. Insulin-like growth factors, IGF-1 and IGF-2, secreted by visceral or mammary adipose tissue have significant paracrine and endocrine effects. These effects can be exacerbated by increased steroid hormone production. Structural studies elucidate how each of the three ligands, insulin, IGF-1, and IGF-2, interacts differently with isoforms A and B of the insulin receptor and with type I IGF receptor and explain how these protagonists contribute to diabetes-associated cancer. The above should inform appropriate treatment of cancers that arise in obese individuals and in those with diabetes mellitus type 2. Novel drugs that target the insulin and insulin-like growth factor signal transduction pathways are in clinical trial and should be effective if appropriate biomarker-informed patient stratification is implemented.

## 1. Introduction

The twentieth century was notable for the eradication of epidemics that had hitherto obliterated large numbers of mankind. This success was realised through widespread vaccination, the introduction of effective antibiotics, and improved nutrition as a result of the implementation of intensive farming.

Tragically, a new epidemic will guarantee the twenty-first century an ignominious place in the history of medicine. As the early twenty-first century has unfolded, obesity in the developed world has increased dramatically. While many are aware of the association between obesity and diabetes because it is reported widely and discussed in the media, few appreciate the associations between obesity-associated diabetes and cancer.

This review summarises the biochemistry behind the physiological response to increased calorie consumption in the absence of increased calorie expenditure and the pathological progression through metabolic syndrome to diabetes mellitus type 2. We examine the evidence for the associated increase in malignancies and why and how these malignancies develop and progress. We focus in particular on the roles of insulin, insulin-like growth factors (IGFs), and the influence of steroid hormones. We conclude with a discussion of how knowledge of the biochemical basis of adiposity- and diabetes-induced cancers should inform the development and selection of effective drugs with which to treat cancer patients with a history of adiposity, metabolic syndrome, and diabetes.

## 2. The Twenty First Century Epidemic

### 2.1. Obesity

Our ancestors have roamed the earth for some four to six million years but obesity has become endemic only within the last thirty years [1]. The World Health Organisation estimates that at present one billion people are overweight worldwide and that at least 300 million are obese. The prevalence of obesity continues to rise at an alarming rate: 26.1% of adults in England are already obese and it is predicted that 60% of adult men, 50% of adult women, and 25% of children will be clinically obese by 2050 [2].

Obesity is defined most commonly using body mass index (BMI) which is calculated as an individual's weight in kilograms divided by their height in meters squared. BMI ranges from overweight (BMI 26–30 kg/m^2^) through class I (BMI 30–35 kg/m^2^), class II (BMI 35–40 kg/m^2^) to class III (BMI > 40 kg/m^2^) obesity. The merit of BMI as a measure of obesity is a matter of debate for several reasons but mainly because it takes no account of body fat distribution. In addition, BMI does not differentiate between the relative contributions to body mass from fat, muscle, or bone. The BMI measure overestimates fatness in individuals with a high muscle mass and underestimates fatness of those with a low muscle mass.

The cost of obesity in personal and socioeconomic terms is huge. Obesity increases the risk of a number of conditions including heart disease, stroke, osteoarthritis, sleep apnoea, and gout. Moreover, obesity is a powerful risk factor for diabetes mellitus type 2; it has been estimated that 80% of patients with diabetes mellitus type 2 are overweight or obese.

### 2.2. The Importance of Insulin

The pioneering work of Banting et al. [3] led to the discovery of insulin and identification of its pivotal role in glucose homeostasis and metabolism through stimulation of increased glucose uptake by cells and enhanced conversion of glucose into glycogen for storage.

**Figure 1:**
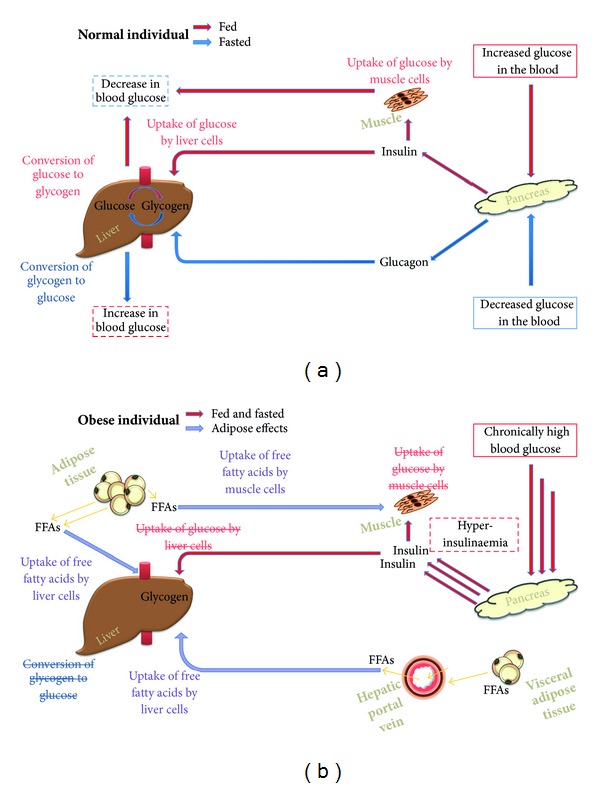
Insulin homeostasis in normal and obese individuals. In normal individuals, the postprandial increase in blood glucose concentration stimulates the beta cells in the Islets of Langerhans of the pancreas to release insulin (a). Insulin stimulates the uptake of glucose by liver and muscle cells and conversion of glucose to the energy store glycogen primarily in the liver and in skeletal muscle. The decrease in glucose levels in the blood of fasted individuals stimulates the alpha cells of the Islets of Langerhans in the pancreas to release glucagon. Glucagon stimulates the conversion of glycogen to glucose by glycogenolysis. In obese individuals, adipocytes in adipose tissue release nonesterified or free fatty acids (FFAs) energy into the circulation (b). The hepatic portal vein provides a direct conduit of free fatty acids from visceral adipose tissue to the liver. The high serum concentrations of nonesterified fatty acids force tissues to prioritise their oxidation as an energy source which prevents glucose uptake. Hence, the obese individual is resistant to the high levels of insulin secreted after a meal and blood glucose levels remain chronically high regardless of the fed state of the obese individual. The pancreas continues to secrete insulin in response to the high blood glucose levels. The obese individual manifests hyperglycaemia and hyperinsulinaemia.

Insulin has acute metabolic effects, the most important of which is to reduce blood sugar levels. Glucose levels increase following a meal, and these high postprandial blood sugar levels trigger release of insulin from the beta cells of the islets of Langerhans in the pancreas (Figure 1). In insulin-target tissues, insulin stimulates translocation of the high affinity GLUT4 glucose transporters from intracellular storage sites to the plasma membrane. GLUT4 then transports glucose into the muscle and liver cells which lowers blood sugar levels. Higher intracellular glucose concentrations stimulate increased glycolysis, glycogen synthesis and fatty acid synthesis, decreased gluconeogenesis, glycogenolysis, and fatty acid oxidation. In fasted individuals, the pancreas secretes glucagon which mobilises stored energy by stimulation of glycogenolysis in its target tissues and, in extreme situations, by neoglycogenesis from amino acids.

### 2.3. Hyperinsulinaemia and the Metabolic Syndrome

**Figure 2:**
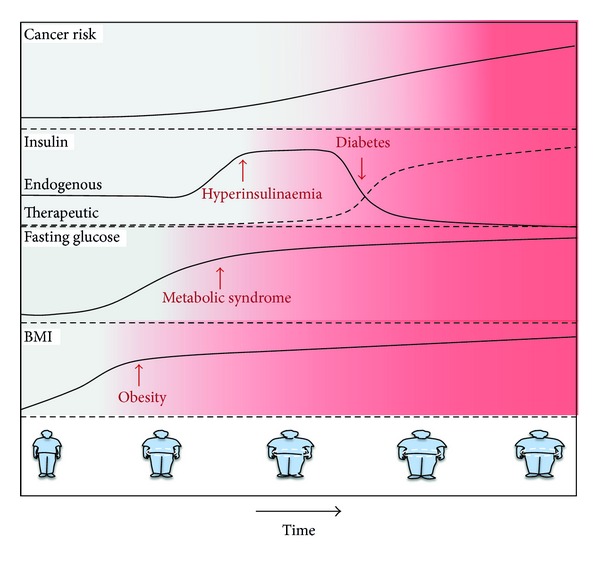
Progression from obesity through metabolic syndrome to diabetes and to increased cancer risk. As an individual's weight increases, they progress from having a normal weight to being overweight and with time become obese. There is a concomitant increase in high levels of circulating nonesterified fatty acids accompanied by high fasting serum glucose concentrations or hyperglycaemia. Many of these obese individuals exhibit a sufficient number of the defining symptoms to be classified as having metabolic syndrome. The high fasting serum glucose and development of insulin resistance induce chronic secretion of insulin from the pancreas, and the individual manifests hyperinsulinaemia. Eventually, the excessive demands on the pancreas lead to failure of insulin secretion, and diabetes is established. Diabetic therapies stimulate insulin secretion or administer exogenous insulin. Insulin concentrations must reach supraphysiological levels to be effective because of the chronic insulin resistance caused by the continued high concentrations of nonesterified fatty acids released from adipocytes. The combination of hyperinsulinaemia and endocrine or paracrine stimuli from adipose tissue increases the risk that these individuals will develop certain types of cancers.

Obesity develops when energy intake exceeds energy expenditure for a prolonged time. In response, the body lays down triglycerides as an energy store in adipose tissue. Excess adipose tissue releases abundant quantities of nonesterified fatty acids which reach supraphysiological concentrations in the serum. The high serum concentrations of these fatty acids force liver, muscle, and other tissues to prioritise oxidation of fats for energy. As a result, the liver and muscle cells do not absorb, store, and metabolise glucose in response to insulin stimuli (Figure 1). Sustained calorie intake results in continuously high serum glucose concentrations, chronic hyperglycaemia, in the majority of obese individuals. Absence of a reaction to insulin secreted by the pancreas in response to the high blood glucose levels means that the obese individual manifests resistance to insulin. The development of this insulin resistance alongside obesity has been classified as metabolic syndrome (Figure 2). When the fat storage capacity of adipocytes is exceeded in obese individuals, skeletal muscle, liver and pancreatic *β*-cells are induced to absorb free fatty acids from serum and to store them as excess fat which drives further fatty acid oxidation in these cells [4, 5]. Resultant intracellular metabolites of triglyceride metabolism in the skeletal muscle and liver cells cause additional insulin resistance.

The metabolic syndrome was identified first in 1988 by Reaven when he noticed that certain individuals had a collection of risk factors for cardiovascular disease, namely, dyslipidaemia, hypertension, and hyperglycaemia [6]. Reaven termed this collection of risk factors Syndrome X. Subsequently called metabolic syndrome, it was defined in 2001 by the Adult Treatment Panel III [7]. They advised that for a diagnosis of metabolic syndrome, an individual should have above a threshold level of three of the following five characteristics: waist circumference, serum triglyceride concentration, serum high-density lipoprotein (HDL) concentration, blood pressure, and fasting serum glucose level (Table 1).

### 2.4. Diabetes Mellitus Type 2 Arises in Obese Individuals

The metabolic syndrome is a biochemical state that is often present in the progression from obesity to diabetes. The combination of reduced glucose metabolism and insulin-resistance leads to constant hyperglycaemia in the fasting as well as in the postprandial state. The pancreas secretes increasingly larger amounts of insulin in an attempt to reduce the high circulating levels of glucose which results in higher than normal serum levels of insulin, hyperinsulinaemia. With time, this excessive insulin production takes its toll on the pancreas and there is a gradual decline in insulin production (Figure 2). Hyperglycaemia in obesity with the resultant continuous insulin production induces metabolic stress in pancreatic *β*-cell mitochondria in the islets of Langerhans. This stress results in the production of reactive oxygen species, which damage the mitochondria. As a consequence, there is decreased *β*-cell mitochondrial ATP production and a reduction in the amount of insulin produced. As well as *β*-cell dysfunction, there is a loss in *β*-cell mass. Eventually, the failing mitochondria are no longer able to support the cell cycle, and apoptosis is induced [8]. Chronic hyperglycemia and hyperlipidemia harm directly pancreatic *β*-cells by two processes referred to as glucotoxicity and lipotoxicity, respectively. When the *β*-cells are exposed continuously to elevated levels of glucose and fatty acids, these processes lead to an inhibition of glucose-induced insulin secretion, impairment of insulin gene expression, and *β*-cell apoptosis [9, 10]. Ectopic storage of triglycerides in pancreatic *β*-cells contributes also to their dysfunction and induces apoptosis [4, 5].

In the end stages of insulin resistance, when an individual has developed insulin insufficiency, their inability to control normal biochemical glucose levels becomes irreversible. The obese individual has now developed chronic diabetes mellitus type 2 known formerly as noninsulin-dependent diabetes or adult-onset diabetes.

Diabetes means “to pass through” and refers to the symptoms of polydypsia and polyuria which are common symptoms in diabetic patients. More water is passed in the urine due to the high concentration of glucose in the urine. Patients become dehydrated and consequently drink more. The word mellitus means honey sweet and refers to the sweetness of the urine that results from the presence of glucose. Diabetes mellitus is a chronic disease of uncontrolled hyperglycaemia, which is secondary to defects in insulin secretion, the action of insulin, or both. For a diagnosis of diabetes mellitus in a patient, confirmation of chronic hyperglycaemia is required [11, 12]. Diabetes mellitus can be classified into type 1, type 2, gestational, and other specific types. Diabetes mellitus type 1 develops as a result of autoimmune or idiopathic destruction of the beta cells of the pancreas. It is diagnosed most commonly in the young. Diabetes mellitus type 2 develops due to metabolic stress-induced beta cell dysfunction or apoptosis as described above. Gestational diabetes is a diagnosis of any glucose intolerance detected in pregnancy. The last rare category is caused by other diseases or specific mutations [12].

Diabetes mellitus type 2 is the most common cause of diabetes mellitus and accounts for 90% of cases. The incidence has risen and continues to rise at a rapid rate. It is estimated that there will be well over 430 million people with diabetes worldwide by 2030 compared with 30 million in 1985 and 285 million in 2010.

The World Health Organisation has reported that diabetes has become a global epidemic. This review focuses exclusively on diabetes mellitus type 2, and this type of diabetes will be referred to simply as diabetes for most of the remainder of the review.

### 2.5. Treatment of Diabetes Mellitus Type 2

An unfortunate consequence of diabetes treatment, which results in part from the insulin resistance of patients, is chronic or transient hyperinsulinaemia (Figure 2). Treatment of diabetes is required to control symptoms and to prevent or slow progression and consequent organ damage. The overriding aim is to prevent or reduce hyperglycaemia. Diabetes treatments can be divided into those that increase endogenous insulin secretion, those that decrease insulin resistance, those that decrease glucose uptake from the intestine, and simple administration of exogenous insulin.

Secretagogues are a class of drugs which act to increase endogenous insulin production from the pancreatic beta cells. They include sulphonylureas and meglitinides. Patients treated with secretagogues are at risk of hypoglycaemia due to overproduction of insulin [13].

Biguanides and thiazolidinediones increase insulin sensitivity in peripheral cells. The exact mechanism of action of the thiazolidinediones is unknown but they increase insulin sensitivity through direct and indirect effects on muscle and adipose tissue [14]. Metformin is a biguanide and is the first-line therapy for the treatment of diabetes mellitus type 2. The therapeutic effects of metformin are understood only partially. Metformin activates adenosine monophosphate- (AMP-) activated protein kinase (AMPK), which leads to suppression of gluconeogenesis in the liver and increased peripheral uptake of glucose by skeletal and adipose tissues. It reduces glucose absorption from the intestine. In addition, metformin increases the affinity of insulin for the insulin receptor, which reduces insulin resistance [14].

Acarbose is an alpha glucosidase inhibitor which acts in the intestinal brush border to prevent the breakdown of complex carbohydrates to glucose and hence the uptake of glucose [13]. The incretins and the inhibitors of their inactivation are a newer group of antihyperglycemic drugs that slow gastric emptying, increase satiety, and decrease postprandial glucagon secretion [15].

When oral hyperglycaemic treatments are contraindicated or cease to be effective in patients with diabetes mellitus type 2, exogenous insulin is given by intramuscular injection. Relatively large doses of exogenous insulin are required to overcome the insulin resistance of the peripheral cells [16]. As a result, although diabetes in these patients was caused by insulin resistance and failure of insulin production as a direct result of hyperinsulinaemia, treatment with exogenous insulin causes necessarily hyperinsulinaemia [16].

### 2.6. Obesity- and Diabetes-Associated Cancer Risk

An estimated 17,294 cases, equivalent to 5.5% of new cancer cases, occurred in obese individuals in 2010 in the UK. The numbers are similar for the whole of Europe; 3.2% of new cancers in men and 8.6% of new cancers in women are estimated to be attributed to obesity [2]. In the United States, 4% of new cancers in men, 34,000 cancers, and 7% of new cancers in women, 50,500 cancers, were due to obesity in 2007 (Figure 2) [17]. Direct evidence of the causative impact of obesity on cancer incidence derives from the demonstration that for obese individuals the risk is reduced by successful gastric bypass surgery [18].

The increased risk in the cancers most closely associated with obesity in men is 1.52 for every additional 5 kg/m^2^ for oesophageal, 1.33 for thyroid, and 1.24 for colon and renal. For women, the increased risk for every additional 5 kg/m^2^ is 1.59 for endometrial, 1.59 for gall bladder, 1.51 for oesophageal, and 1.34 for renal cancer (Table 2). Associations are found for a number of other cancers including pancreas and breast and colon cancer in women [19]. These cancer types account for 65% of all new cancers related to obesity [2, 20, 21]. The percentage of cases attributed to obesity is as high as 40% for oesophageal and endometrial cancers.

Diabetes is associated with increased risk of certain types of cancer [22–24] (Figure 2). The increased relative risk for diabetic individuals compared to nondiabetics is 2.5-fold for liver, 2.22-fold for endometrial cancer, 1.5–2.0-fold for pancreatic cancer and non-Hodgkin's lymphoma, and 1.2–1.5-fold for biliary tract, renal, bladder, breast and bladder cancer, and oesophageal adenocarcinoma (Table 2). There is an increased risk in men with diabetes for oesophageal adenocarcinoma but a reduced risk for oesophageal squamous cell carcinoma [25]. There is no or inconclusive evidence for other cancers apart from prostate cancer for which there is a reduced risk [26].

Although the increased risks associated with obesity and diabetes may appear relatively small for some cancers, they translate into a significant number of new cancer cases. Across 30 European countries, obesity accounts for more than 124,000 new cancers per year [27], all of which are potentially avoidable.

### 2.7. Obesity- and Diabetes-Associated Cancer Progression

Intuitively, given its role as a risk factor and the putative mechanisms involved, obesity might be expected to be associated with worse prognosis in cancer patients. The evidence from the majority of studies that have addressed this issue is persuasive. In pancreatic cancer, it has been shown that increased intra-abdominal fat is associated with shorter survival [28].

For colon cancer, some studies have found no effect but others have found obesity to be an independent prognostic factor associated with a higher mortality, especially in men [29–31]. For endometrial cancer, 90% of women with the most common form of endometrial cancer, type I, are obese [32], and higher BMI is associated with increased mortality [33]. For epithelial ovarian cancer, a meta-analysis of observational studies concluded that obesity is associated with higher mortality [34].

The strongest evidence that obesity is associated with a poor prognosis is for breast cancer [35]. In a 2002 overview, the majority of studies found obesity was associated with increased risk of recurrence and death [36]. A recent large Danish study found that tumours in obese women were larger and were more likely to be of a high histological grade and to have metastasised to lymph nodes [37]. Obese women had a higher risk of distant metastases and death than nonobese women. There is some encouragement that prognosis can be improved by increased physical activity [38, 39].

### 2.8. Types and Roles of Adipose Tissue

The question arises, why do individuals who are obese and those who progress through metabolic syndrome to develop diabetes have an increased cancer incidence and worse prognosis? To answer this question, we must consider the biochemical consequences of obesity. The primary roles of adipose tissue have long been considered to be the storage of energy, thermal regulation, and mechanical protection. More recently, the role of adipose tissue as an endocrine or metabolic organ has been recognised. This section examines the different types of adipose tissue and their impacts on paracrine and endocrine levels of insulin, IGFs, and steroid hormones.

Adipose tissue is a specialized connective tissue made up of different cell types, including preadipocytes, adipocytes, fibroblasts, macrophages, and blood vessels [40], of which the majority are adipocytes. Approximately 15% of the body mass of a male of average weight, 70 kg or 11 stone, is adipose tissue. Adipose tissue is distributed throughout the body. Its percentage of body mass and pattern of distribution in the body are influenced by numerous factors including the sex, age, diet, physical activity level, and genotype of an individual [41]. In the body, lipids, commonly referred to as fat, are present usually in the form of triglycerides and make up 80% of adipose tissue. Although stored largely in adipose tissue, lipids are found in other tissues especially in pathological conditions [41]. Triglycerides are present also in the plasma. The body adapts to the accumulation of increased levels of triglycerides by hyperplasia and hypertrophy of its adipose tissue [42].

Adipose tissue can be defined by its biochemical role [41]. Brown adipose tissue is highly metabolic and utilises a large amount of glucose. Accordingly, the majority of the adipocyte cell volume is occupied by large spherical mitochondria [40], and it is this feature which gives rise to the brown colour. Brown adipose tissue produces heat through a specialised metabolic pathway which mobilises stored energy by breaking down triglycerides to generate heat energy. Brown adipose tissue is abundant in small animals and newborns and has been shown to have an important role in thermal homeostasis in adults.

White adipose tissue is a lipid-rich tissue that has been considered traditionally as an energy store of excess triglycerides that will be mobilised to release fatty acids when the body requires more fuel. The discovery of its ability to function as an endocrine and paracrine organ indicates that the metabolic role of white adipose tissue is more complex than appreciated previously [43]. Unlike brown adipocytes, white adipocytes are spherical cells that contain a single lipid droplet which accounts for 90% of the cell volume [40].

The mammary gland contains specialized adipose tissue that is important in epithelial cell growth and milk production while bone marrow adipose tissue is known to have a role in osteogenesis and haematopoiesis [41].

Alternatively, adipose tissue can be classified by its anatomical position which dichotomises it into subcutaneous and internal or visceral adipose tissues. Subcutaneous adipose tissue is the adipose tissue that accumulates beneath the skin. The term “viscera” refers to “organs in the cavity of the body” [41]. Internal or visceral adipose tissue encases these body organs.

### 2.9. Pathological Effects of Different Types of Adipose Tissue

There is increasing evidence that the anatomical position of adipose tissue determines the effects that the adipose tissue has on an individual and predicts the associated morbidity from cardiovascular disease and diabetes and eventually cancer [44]. This realisation has led to revised measures of obesity in addition to BMI. Preferred methods acknowledge the relative contributions in obese individuals of different anatomical types of adipose tissue.

Waist-to-hip ratio (WHR) is used widely to measure regional adipose tissue distribution because a relatively large waist measurement indicates that an individual has an excess of visceral adipose tissue. WHR has been shown to be a better predictor of subsequent cardiovascular disease and diabetes than BMI or skin fold thickness [45]. An increasing number of epidemiological studies use WHR or waist circumference as more useful measures of obesity [46]. It must be remembered that waist circumference will not be due exclusively to visceral adipose tissue but also to abdominal subcutaneous adipose tissue.

Visceral adipose tissue has been shown to have a greater influence on the development of hypertension and cardiovascular disease, and of insulin resistance and diabetes, than subcutaneous adipose tissue [44, 45]. A study on insulin sensitivity in obese adults showed that when the data was adjusted for BMI and visceral adipose tissue, subcutaneous adipose tissue was protective against development of insulin resistance. When the data was adjusted for BMI and subcutaneous adipose tissue, increased visceral adipose tissue was associated with an increase in insulin resistance. The implication is that visceral adipose tissue promotes insulin resistance but subcutaneous adipose tissue does not [47].

Multiple reasons have been postulated to explain the causal effects of visceral adipose tissue on the development of insulin resistance and cancer. When abdominal visceral adipose tissue, which is positioned around the liver, enters a hyperlipolytic state and releases free fatty acids, they travel through the portal vein to the liver (Figure 1(b)). The resultant high levels of free fatty acids in the liver impair hepatic metabolic function which leads to insulin resistance, hyperinsulinaemia, and hypertriglyceridaemia [48] which in turn contribute to the development of the metabolic syndrome. Molecules released by other adipose tissue do not travel directly to the liver. Inflammatory cells are present in abundance in visceral adipose tissue and the secretion of inflammatory mediators into the body creates a chronic inflammatory state which is thought to generate a protumourigenic environment [49].

The distribution of adipose tissue may explain partially the variance in the development of the metabolic syndrome and different types of cancer between men and women and between different age groups. Visceral adipose tissue increases with age and weight in both sexes but the progression is more complicated in females. Overall, females have a greater percentage body mass of adipose tissue than males but in premenopausal women, adipose tissue is predominantly subcutaneous; this is true in obese and in lean individuals [45]. In men, the majority of adipose tissue is abdominal visceral adipose tissue. This predominance of visceral adipose tissue increases with age in a linear fashion. Men have been shown to have 48% more adipose tissue around the waist than premenopausal women, of which the majority is visceral adipose tissue. There is evidence of this sexual dimorphism in fat patterning even in prepubertal children but the difference is most marked in young adolescents [50]. The distribution of adipose tissue changes in postmenopausal women, with an accumulation of abdominal adipose tissue. It has been found that postmenopausal women have 49% more visceral adipose tissue than premenopausal women [51].

### 2.10. Endocrine Role of Adipose Tissue

The role of adipose tissue as an endocrine tissue is relevant to the development of the metabolic syndrome, insulin resistance, diabetes, and cancer. Adipose tissue has been shown to produce adipokines, including leptin and adiponectin. Leptin is involved in the maintenance of normal body weight. Leptin binds the leptin receptor in the hypothalamus [52, 53] which results in appetite suppression and increased energy expenditure. Obese individuals become leptin resistant, and the increased production of leptin in response to raised food consumption does not stimulate reduced energy intake or increased energy expenditure [53, 54]. Elevated levels of circulating leptin are associated with an increased risk of colorectal [55], endometrial [56], and in some studies, breast cancer [57]. Increased risk is thought to occur through direct activation of leptin receptors to induce cell proliferation and reduce apoptosis [58, 59]. It has been reported that leptin can influence IGF signal transduction [60] which could modulate the effects of IGFs on cancer risk and progression that are described below.

Adiponectin acts to sensitive cells to insulin via interaction with the AdipoR1 and AdipoR2 receptors. Activation of the receptors has also anti-inflammatory effects and increases fatty acid oxidation which prevents insulin resistance [60]. Whilst the factors that control adiponectin levels are not defined clearly, levels are lower in obese individuals and higher levels can be achieved through weight loss [61]. Low levels of adiponectin are associated with an increased risk of breast, endometrial, prostate, colorectal, and renal carcinomas [62–65].

Adipose tissue produces also tumour necrosis factor-*α*, interleukin-6, angiotensinogen, lipoprotein lipase, plasminogen activator inhibitor-1, monocytic chemoattractant protein, oestrogens, aromatase, IGFBP-3, IGF-1, and IGF-2 [45]. All of these molecules may have a role in the complex development of cancer in obesity. In this review, we concentrate on the production of IGF-1, IGF-2, IGFBP-3, oestrogens, 17*β*-hydroxysteroid dehydrogenase, and aromatase.

### 2.11. Adiposity and Insulin-Like Growth Factors

**Figure 3:**
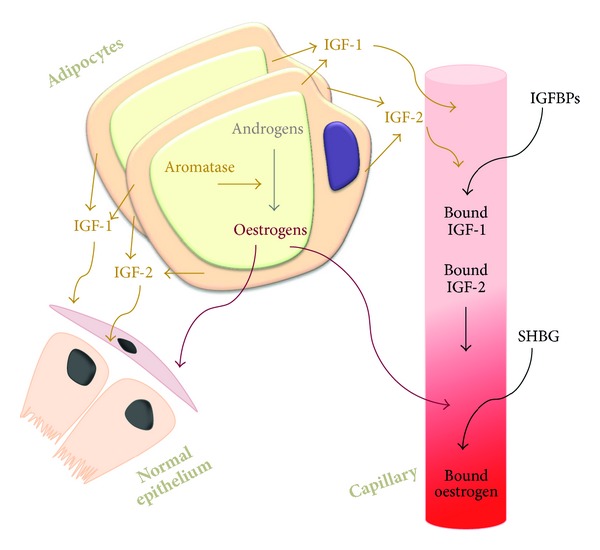
Adipose tissue as an endocrine organ. Adipocytes express enzymes involved in steroid metabolism, notably 17*β*-hydroxysteroid dehydrogenase and aromatase which converts androgens into oestrogens. There is a resultant increase in paracrine concentrations of oestrogens and a significant increase in the concentration of circulating oestrogens in obese men and in obese postmenopausal women compared to nonobese individuals. In the blood, oestrogen is complexed with sex hormone-binding globulin (SHBG). Adipocytes secrete several protein hormones including IGF-1 and IGF-2. IGF-1 and IGF-2 enter the blood stream where they can be bound by insulin-like binding proteins (IGFBPs). Local concentrations of IGF-1 and IGF-2 may be particularly high and are unlikely to be reduced by feedback mechanisms.

Numerous studies have shown that adipocytes produce IGF-1 and IGF-2 (Figure 3). This production was demonstrated first in animal studies. For instance, both IGF-1 and IGF-2 mRNAs are synthesised in rat and porcine adipose tissues [66, 67]. Plasma IGF-2, but not IGF-1 or IGFBP-3, was associated positively with back fat depth [68]. Free and total IGF-1 concentrations measured in serum by enzyme-linked immunosorbent assays were increased in patients with visceral obesity compared with nonobese individuals [69]. *In vitro *models in which human adipocytes were cultured in chemically defined culture conditions, demonstrated IGF-1 production from adipocytes and preadipocytes; production was tenfold higher from adipocytes than from preadipocytes.

Some studies have shown that there are lower serum IGF-1 concentrations in obesity possibly due to the negative feedback that the initial rise in serum IGF-1 has on the production of growth hormone (GH) by the pituitary gland [70]. A decrease in free GH would reduce stimulation of hepatocyte production of IGF-1. Although the total levels of circulating free IGF-1 in the body may be decreased, it is probable that IGF-1 levels in the adipose tissue remain higher than normal as a result of the increased production secondary to adipocyte hyperplasia and hypertrophy. These local concentrations of IGF-1 may exert significant effects on surrounding cells and adjacent tissues via paracrine effects rather than on distant tissue via endocrine mechanisms (Figure 3). Such local effects could contribute to the observed greater contribution of visceral adipose tissue to cancer risk than overall adipose tissue.

### 2.12. Adiposity and Steroid Hormone Production

**Figure 4:**
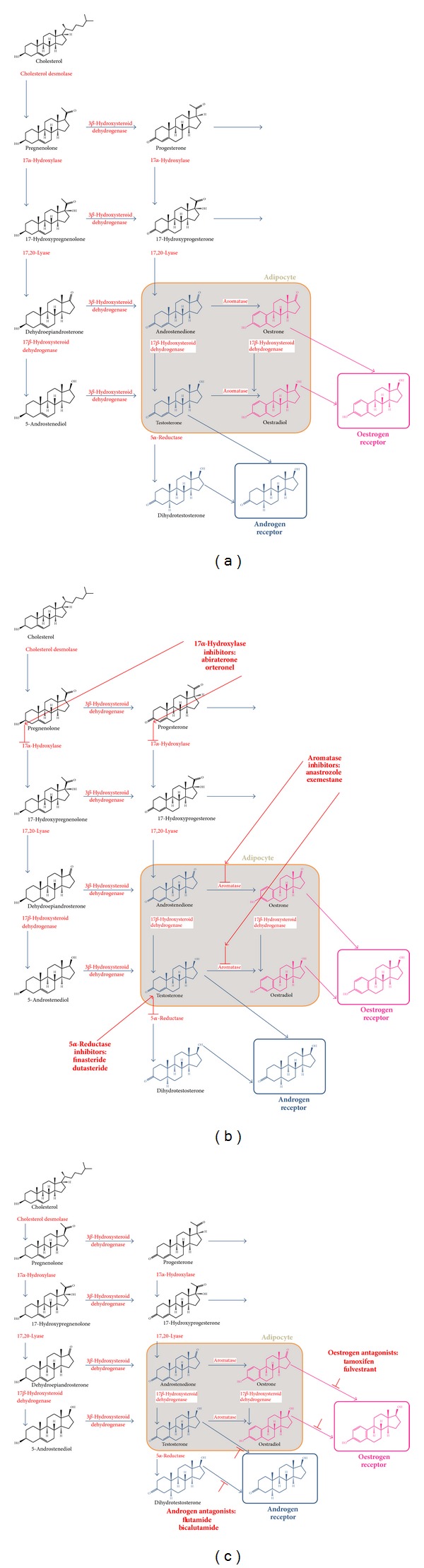
Steroid hormone biosynthesis and therapeutic prevention of steroid hormone action. The sterol cholesterol which contains 27 carbons and includes four interconnected cyclic hydrocarbons is the precursor of all steroid hormones (a). Removal of the cholesterol side chain yields the 21-carbon pregnenolone which is metabolised further to give active progestogens which are in turn converted into glucocorticoids and mineralocorticoids (not shown). Pregnenolone and progesterone may be converted also to 17*α*-hydroxy pregnenolone and 17*α*-hydroxy progesterone which are the precursors of the 19-carbon androgens. Metabolism of androgens by aromatisation of the first cyclic hydrocarbon and removal of carbon 19 produces the 18-carbon oestrogens. Two important enzymes in the end stages of androgen and oestrogen synthesis are expressed in adipocytes: 17*β*-hydrosteroid dehydrogenase converts androstenedione into the much more potent testosterone and oestrone into the more potent oestradiol. Aromatase converts the androgens androstenedione and testosterone into oestrone and oestradiol, respectively. All steroid hormones interact with and activate a cognate receptor which is a ligand-dependent transcription factor. For instance, all active androgens interact with the androgen receptor and all oestrogens interact with an oestrogen receptor. Systemic therapeutic intervention to inhibit androgen or oestrogen action involves inhibition of enzymes involved in their synthesis (b) or competitive inhibition of the interaction of the steroid ligands with their receptors (c). Recently, 17*α*-hydroxylase inhibitors such as abiraterone and orteronel have been developed and are in clinical trial for the treatment of antiandrogen refractory prostate cancer. Inhibitors of 5*α* reductase such as finasteride and dutasteride are used in the treatment of benign prostatic hyperplasia and male pattern baldness. Aromatase inhibitors such as type I inhibitor anastrozole and type II steroid inhibitor exemestane are used widely in the treatment of postmenopausal women with breast cancer. Oestrogen antagonists include the partial antagonist tamoxifen which is a triphenylethylene derivative and the pure antioestrogen fulvestrant which is a steroid. Androgen antagonists include the nonsteroidal pure antiandrogens flutamide and bicalutamide which is the mainstay of systemic therapy in prostate cancer patients.

Adipose tissue contains enzymes that are involved in steroid biosynthesis and is therefore a source of extragonadal oestrogens in men and women (Figure 3). Adipocytes express 17*β*-hydroxysteroid dehydrogenase that converts androstenedione to the more active androgen testosterone and converts oestrone to oestradiol (Figure 4). Aromatase, an enzyme of the cytochrome P450 family, is encoded by the* CYP19* gene [71] and catalyses the final step of oestrogen synthesis by aromatisation of androgens to oestrogens, in particular androstenedione to oestrone and testosterone to oestradiol. Increased adiposity leads therefore to higher local tissue concentrations of oestrone and oestradiol. There is some evidence that inflammation associated with adiposity increases aromatase activity [72].

In premenopausal women, aromatase is expressed in ovarian granulosa cells, placental syncytiotrophoblast cells, brain, breast cancer, skin fibroblasts, bone osteoblasts, chondrocytes, and adipose stromal fibroblasts [73]. Peripheral aromatisation is particularly relevant in postmenopausal women following cessation of androgen and oestrogen synthesis in the ovaries. In these women, circulating oestrogens are derived principally by peripheral aromatisation of androgens produced in the zona reticularis of the adrenal cortex.

A detailed study of the relationship between BMI and serum oestrogen levels in postmenopausal women showed an increase in all measures of oestrogen but a most marked increase in free oestrogen with increasing BMI. Serum concentrations of the most potent oestrogen, oestradiol, were twofold higher in the upper quartile compared to in the lower quartile, 57.9 pM compared to 29.9 pM, and serum oestrone concentrations were 1.5-fold higher, 133 pM compared to 89 pM. The level of oestrogens increases in obese women due to the greater amount of adipose tissue and hence aromatase activity. Obesity causes also a reduction in sex hormone-binding globulins (Figure 3) and hence there is more free circulating oestrogen in obese individuals [74]. Free serum oestradiol was 2.5-fold higher in the upper quartile compared to in the lower quartile, 29 pM compared to 12 pM. These free serum oestradiol concentrations are high enough to occupy and activate the oestrogen receptor, and because the increase in oestradiol concentration observed in obese women is in the linear range of response to oestradiol, it would be predicted to increase significantly the induction of gene expression and cell proliferation in oestrogen-responsive malignant cells [75–78]. In breast cancer cells, induction of the expression of thirteen genes was half-maximal between 20 and 50 pM oestradiol and the increase in oestradial-stimulated cell proliferation was half-maximal at 30 pM oestradiol [77, 78]. Local tissue concentrations of oestradiol in obese individuals will exceed most probably those measured in serum (Figure 3).

## 3. The Major Molecular Pathways Involved

Several mechanisms have been postulated to explain the pathophysiology behind the obesity and diabetes-associated increased risk and progression of cancer. Insulin, IGFs and their receptors have all been suggested to play an important role. To understand how these different stimuli might influence cancer development and progression both individually and in concert, it is necessary to consider the biochemical basis of their activity.

### 3.1. Insulin

**Figure 5:**
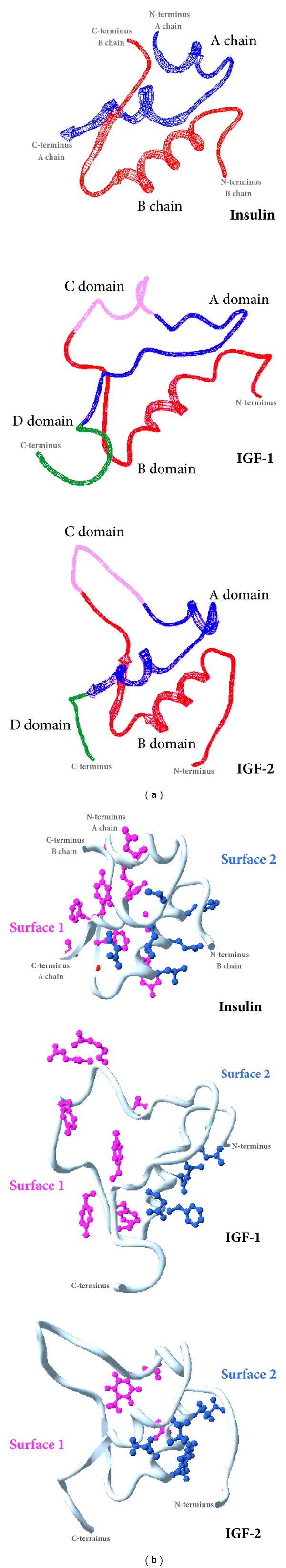
Representation of the three-dimensional structures of insulin, IGF-1, and IGF-2, and of the two binding surfaces on the ligands. Ribbon representations of the three-dimensional structures of the three ligands are shown with the insulin B chain and equivalent B domains of IGF-1 and IGF-2 coloured in red, the C domains of IGF-1 and IGF-2 in pink, the insulin A chain and A domains of IGF-1 and IGF-2 in blue, and the D domains of IGF-1 and IGF-2 in green (a). The three insulin *α* helices, one in the B chain and two in the A chain, are visible as are the equivalent structures in IGF-1 and IGF-2. The positions and orientations of the side chains of the residues implicated in interaction of the ligands with the receptors are shown on the backbone of the molecule structures which are coloured in grey (b). The side chains are shown in ball-and-stick view. The side chains of the residues located on binding Surface 1 are coloured in pink and those located on binding Surface 2 are coloured in blue. Residues ValB12, TyrB16, GlyB23, PheB24, PheB25, TyrB26, GlyA1, IleA2, ValA3, GlnA5, TyrA19, and AsnA21 of insulin are involved in Surface 1 and HisB10, GluB13, LeuB17, SerA12, LeuA13, and GluA17 in Surface 2. For IGF-1, residues Phe23, Tyr24, Tyr31, Arg36, Arg37, Val44, Tyr60, and Ala62 form Surface 1 and residues Glu9, Asp12, Phe16, Leu54, and Glu58 are thought to contribute to Surface 2. In IGF-2, residues Val14, Phe28, and Val43 are included in Surface 1 and residues Glu12, Phe19, Leu53, and Glu57 in Surface 2.

As described above, insulin is synthesised in and then secreted from beta cells of the pancreatic islets of Langerhans in response to plasma glucose. Insulin was the first protein to be sequenced and one of the first to have its structure solved using X-ray crystallography. Insulin is synthesised as a precursor protein, proinsulin, and is processed by the removal of a central C peptide which lies between the B and A polypeptide chains. Insulin comprises two polypeptide chains: an A chain of 21 amino acid residues that contains two regions of *α*-helix and a B chain of 30 residues that contains a region of *α*-helix towards the N-terminus (Figure 5(a)). The two chains are linked together by two interchain disulphide bridges. There is a third disulphide bridge within the A chain between the sixth and eleventh residues.

### 3.2. Insulin-Like Growth Factors

The pioneering work of Salmon Jr. and Daughaday [79] led to the discovery of IGF-1 as the first known mediator of the effects of growth hormone. IGF-1 is synthesised by the liver under the control of growth hormone and stimulates bone growth because it increases the proliferation of cartilage cells in the epiphyseal plate of long bones. IGF-1 contains 70 amino acid residues in a single polypeptide chain and has three intramolecular disulphide bonds congruent to those found in insulin. It contains the *α*-helix in the same location as that in the B chain of insulin but the 2 stretches of *α*-helix in the equivalent of the A chain of insulin are less well defined (Figure 5(a)). It retains the equivalent of the C peptide of insulin and has an additional sequence, the D domain, at the carboxy-terminus. IGF-2 was discovered at about the same time as IGF-1 [80]. It is closely related to IGF-1 and is produced primarily by the liver, but its production is not controlled by growth hormone. IGF-2 expression is controlled by methylation of a differentially methylated region located upstream of the *IGF-2* gene. IGF-2 is expressed normally from the paternal chromosome only as a result of maternal genetic imprinting [81]. Loss of imprinting leads to overexpression of IGF-2 and occurs frequently in human cancers [82, 83]. IGF-2 contains 67 residues in a single polypeptide chain, retains a C- and D-domain, and has a similar structure to those of insulin and IGF-1 with three congruent intramolecular disulphide bonds.

The boundaries between insulin and IGF signal transduction were thought originally to be quite distinct. It has become apparent that the structural similarities between insulin, IGF-1, and IGF-2 are sufficiently strong to allow them to interact with each other's receptors. Further, their cognate receptors are closely related and activate many common intracellular signal transduction molecules.

### 3.3. The Insulin Receptor

The cellular effects of insulin are mediated by the insulin receptor. The insulin receptor has a highest affinity for insulin and a lower affinity for the two related growth factors, IGF-1 and IGF-2 (Table 3). The insulin receptor is a cell surface tyrosine kinase receptor of 320 kDa. The receptor is present in its unbound form in the plasma membrane as a disulphide-linked heterotetramer which is referred to usually as the insulin receptor dimer.

**Figure 6:**
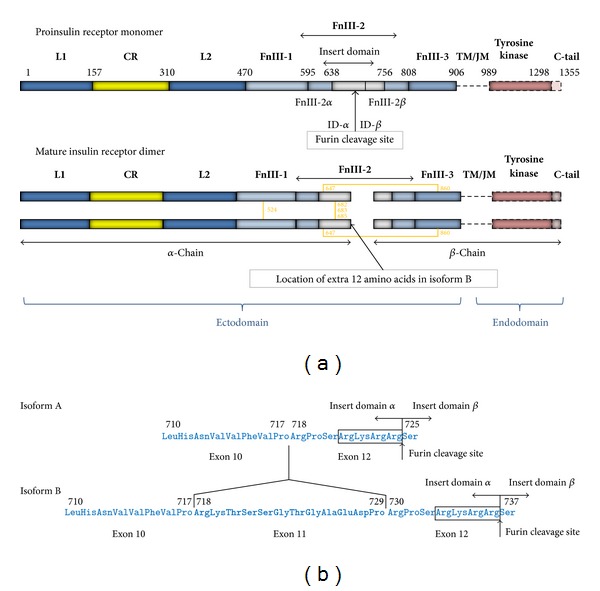
Representation of the domain structure of the human insulin and type I IGF receptors. The insulin receptor and type I IGF receptor are synthesised as single polypeptide chains that comprise several congruent, distinct domains. A linear representation of the domain structure of the proinsulin receptor isoform B after the removal of the signal peptide is shown above, a representation of the homodimer (a). From the amino-terminus, the individual domains are coloured as follows: leucine-rich repeat domain 1 (L1, blue), a cysteine-rich region (CR, yellow), a second leucine-rich repeat domain (L2, blue) followed by three fibronectin type III domains (FnIII-1, FnIII-2, and FnIII-3, different shades of pale blue). FnIII-2 is interrupted by a ~120 residue insert domain (ID, grey) that contains the furin proteolytic cleavage site which is cleaved by a protease to create the *α* and *β* chains of the receptors. The segments of the insert domain that are in the *α* and *β* chains are termed ID*α* and ID*β*, respectively. The single helix that spans the plasma membrane (TM, dashed line) is C-terminal to the FnIII-3 domain and is followed by the intracellular domains comprising a ~40 amino acid residue intracellular juxtamembrane region (JM, dashed line), a tyrosine kinase (TK) catalytic domain, and an ~60 amino acid residue carboxyl-terminal tail. Intramonomer and intermonomer disulphide bonds are shown as ochre coloured connecting lines. The position of the furin cleavage site is indicated below the proinsulin receptor monomer and the position of the extra 12 amino acid residues present in isoform B of the insulin receptor is indicated below the depiction of the mature insulin receptor dimer. The amino acid sequences around the furin cleavage sites in isoform A and isoform B [311] of the insulin receptors are shown (b). The additional 12 residues encoded by exon 11 that are present in isoform B of the insulin receptor are shown in bold. The presence of 12 additional residues, three amino acids from the carboxy-terminus of the *α*-chain, in the *α*-chain insert domain impinges upon Site 1 of the insulin receptor binding pocket [84] and affects the ability of IGF-1 and IGF-2 to interact with the insulin receptor isoform B.

After synthesis, the insulin receptor protein undergoes posttranslational proteolytic cleavage to generate an amino-terminal *α*-chain and a carboxyl-terminal *β*-chain (Figure 6(a)). The two chains are then rejoined covalently by a disulphide bond between the *α* and *β* chains of each monomer. The extracellular part of the insulin receptor dimer, called the ectodomain, contains both *α*-chain and 194 residues of each *β*-chain. The two monomers in the insulin receptor dimer are bound covalently by four disulphide bonds between the two *α* chains.

The crystal structure of the ectodomain of the insulin receptor dimer has been solved [84, 85], and additional knowledge is available from molecular modelling, mutagenesis of both insulin and the insulin receptor, analysis of patients with defective insulin signaling, and thermodynamic and kinetic analyses of the binding reaction [86, 87]. Each insulin receptor monomer ectodomain comprises a leucine-rich repeat domain (L1), a cysteine-rich region (CR), and a second leucine-rich repeat domain (L2) followed by three fibronectin type III domains: (FnIII-1, FnIII-2, and FnIII-3). FnIII-2 is interrupted by a ~120 amino acid residue insert domain (ID) that contains the furin proteolytic cleavage site that is cleaved by a protease to create the *α* and *β* chains (Figure 6(a)). The segments of the ID that are in the *α* and *β* chains are termed ID*α* and ID*β*, respectively. The single helix that spans the plasma membrane is C-terminal to the FnIII-3 domain and is followed by the intracellular domains comprising, a ~40 amino acid residue intracellular juxtamembrane region (JM), a tyrosine kinase (TK, dark salmon) catalytic domain, and an ~100 amino acid residue carboxyl-terminal tail (C-tail, dull pink).

**Figure 7:**
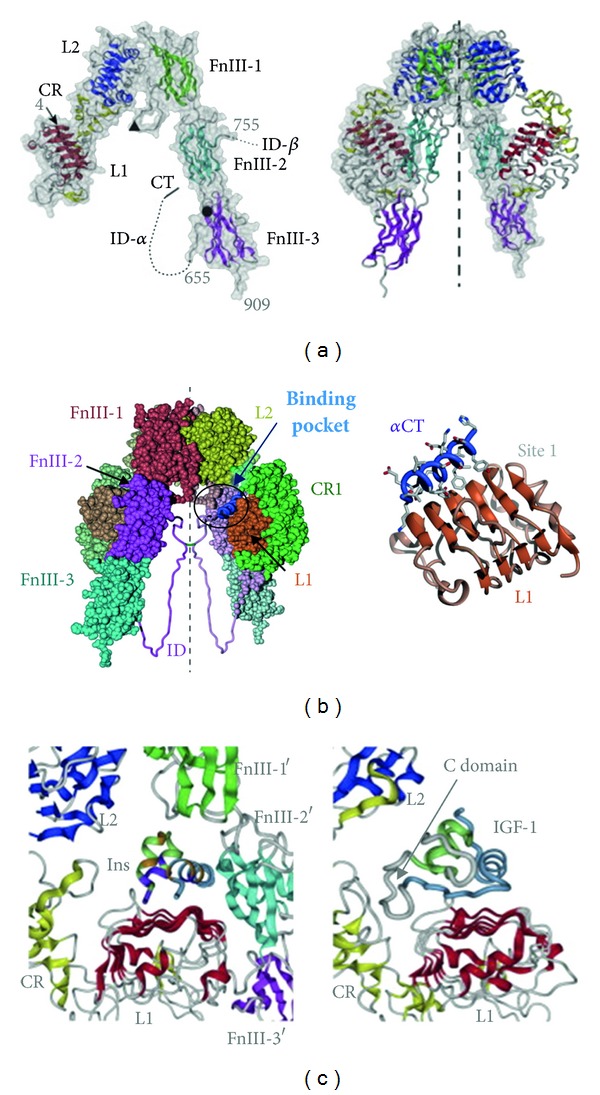
Representation of the crystal structure of the ectodomain of the insulin receptor and model for the interaction of insulin and IGF-1 with their cognate receptors. The three-dimensional structures of a monomer (LHS) and homodimer (RHS) of the insulin receptor isoform B ectodomain are shown (a). Domains are coloured: L1, red; CR, yellow; L2, blue; FnIII-1, green; FnIII-2, cyan; and FnIII-3, magenta. The observed location of the inter-*α*-chain disulphide at Cys524 is indicated by a (▴) symbol, the *α*-*β* chain disulphide at Cys647–Cys860 by a (●) symbol. Images are taken with permission from [312]. The structures of *α*-chain residues 1–3 (*α*-chain N-terminus) and 656–719 (ID-*α*/CT) and of *β*-chain residues 724–754 (ID-*β*) and 910–917 (C-terminus of ectodomain) were not solved. The observed termini of the regions of resolved structure within the insert domain of the *α*-chain and *β*-chain of the ectodomain monomer are numbered. The insulin receptor ectodomain homodimer is illustrated in its apoconformation, with the twofold symmetry axis indicated by a dashed vertical line ((b), LHS) [85]. The first monomer at the front of the depiction is shown in saturated colour and the second monomer in less intense colour. The thin tubes of the insert domains (ID) indicate their speculative paths. The circled region identified by a blue arrow represents one binding pocket. The region contains the juxtaposition of the residues, derived from L1 of the first monomer and the *α*CT helix of the second monomer that contribute to binding Site 1 with the residues, derived from the FnIII-1 and FnIII-2 junction of the second monomer that contribute to Site 2. The Site 1 tandem element, formed by the surface of the central *β*-sheet of L1 of the first insulin receptor monomer and the *α*CT helix of the second insulin receptor monomer, is shown (RHS). Images are taken with permission from [86]. The proposed location of insulin bound in an insulin receptor binding pocket formed between two insulin receptor monomers with Site 1 from mainly one monomer and Site 2′ from the second monomer of the insulin receptor ectodomain [85, 107, 312] is shown in (c) (LHS). Domains of the receptor are coloured as in (a). Colouring of individual C*α* positions within insulin is as follows: residues forming binding Surface 1 of insulin, A1–3, A5, A19, A21, B12, and B16, are shown in purple; residues forming binding Surface 2, A12-13, A17, B13, B17, and B19, are shown in orange; the remaining A-chain residues are in light green and remaining B-chain residues in light blue. Insulin residues B22–B30 are omitted from the model [107]. The proposed location of IGF-1 bound at Site 1 of type I IGF receptor binding pocket is shown (RHS) [313]. The colouring of domains of the type I IGF receptor follows that of the insulin receptor in (a). The backbone of IGF-1 is coloured according to the corresponding colours of the A and B chains of insulin outlined above. The images are taken with permission from [312].

The structure of the extracellular domains of each receptor monomer resembles an inverted “V” of which the L1-CR-L2 domains form one arm and the three FnIII domains form the other. In the receptor dimer, the second monomer is related to the first by a twofold rotation about the axis of the inverted “V” which results in the L1-CR-L2 arm of one monomer being packed against the three FnIII domains of the other (Figure 7(a)).

### 3.4. Interaction of Insulin with the Insulin Receptor

Insulin makes contact with the insulin receptor through two distinct surfaces. Surface 1 approximates to the surface responsible for insulin dimerisation, which is known as the “classic binding surface.” It includes ValB12, TyrB16, GlyB23, PheB24, PheB25, TyrB26, GlyA1, IleA2, ValA3, GlnA5, TyrA19, and AsnA21. Surface 2 overlaps the surface on the insulin dimer responsible for hexamer formation and includes HisB10, GluB13, LeuB17, SerA12, LeuA13, and GluA17 (Figure 5(b)).

The structure of the insulin : insulin receptor complex has not been solved. The current model for insulin interaction with the insulin receptor proposes that there are two equivalent ligand binding pockets in the insulin receptor dimer. Within each pocket, there are two distinct sites of interaction with insulin [88, 89]. The central *β*-sheet of the L1 domain from one receptor monomer and the C-terminus of the FnIII-2 insert domain (*α*-CT) from the second receptor monomer constitute one binding site, Site 1 (Figure 7(b)). The structure of the *α*-CT segment has been resolved recently [84]; residues 693–710 form an *α*-helical structure that is packed against the L1 domain while the remaining 21 carboxy-terminal residues are disordered. Residues at the junction between FnIII-1 and FnIII-2 domains from the second receptor monomer constitute the second binding site, Site 2. High-affinity binding involves interaction of surface 1 of an insulin molecule with Site 1 from mainly one receptor monomer and surface 2 of the insulin molecule with Site 2 from the second receptor monomer; the insulin molecule bridges the two receptor monomers. Each ligand binds the receptor with a stoichiometry of 1 : 1 at physiological concentrations. Whilst the receptor dimer has two identical Site 1-Site 2 binding pockets, insulin molecules cannot occupy both pockets simultaneously. Interaction of a second ligand with the previously unoccupied binding pocket accelerates the dissociation of the first ligand which results in the observed negative-cooperativity binding mechanism. Relatively little conformational change in the receptor structure occurs after high-affinity interaction with ligand [86].

### 3.5. Isoform A of the Insulin Receptor

Evolution has provided mammalian insulin receptors with an additional exon, exon 11, which encodes the 12 amino acids from residues 718–729 until just three residues from the carboxyl-terminus (residue 732) of the *α* chain (Figure 6(b)). The extra amino acid residues are thought to have a disordered structure and are located just carboxy-terminal to the *α*-helical region, which is packed against the L1 domain to form the insulin binding Site 1 of the receptor [86]. In mammals, the insulin receptor can be transcribed without or with exon 11 to produce isoform A or isoform B, respectively. The discovery of the two insulin receptor isoforms as part of the original cloning and sequencing of the insulin receptor mRNA [90] was regarded initially as a molecular curiosity but is now recognised to be of considerable relevance to the role of hyperinsulinaemia and diabetes in cancer risk and cancer progression and in the potential effectiveness of different IGF-targeted therapies.

The two isoforms have different tissue distributions [91]. Isoform B of the insulin receptor is the predominant form in classic insulin target tissues such as liver and skeletal muscle whereas isoform A tends to be expressed in nonclassic insulin target tissues. Importantly, isoform A is the predominant form of the insulin receptor expressed in the foetus and in various types of cancer including breast, colon, lung [92, 93], thyroid [94], prostate [95], and ovary [96]. The ratio of isoform A to isoform B is higher in breast cancer than in normal breast tissue [93]. In thyroid cancer, isoform A is related to differentiation, being expressed at higher levels in less well-differentiated tumours [94]. Isoform A expression is not limited to cancers of epithelial origin and is the predominant isoform in some sarcomas [97] and gestational trophoblastic neoplasias [98].

### 3.6. Interaction of Insulin, IGF-1, and IGF-2 with Isoform A of the Insulin Receptor

Subsequent to the discovery of isoform A, it was recognised that isoforms A and B had differing affinities for insulin with isoform A having a twofold higher affinity than isoform B [99]. The absence of exon 11 in isoform A, however, produces a more promiscuous receptor with reduced specificity for insulin; isoform A has increased affinities for IGF-1 and IGF-2. The affinity of isoform A of the insulin receptor for IGF-1 is about 10-fold higher than the affinity of the B isoform of the insulin receptor, and for IGF-2 it is about 5-fold higher [100, 101]. The affinity of IGF-2 for isoform A of the insulin receptor approaches its affinity for type I IGF receptor (Table 3) [92, 100–102]. Although the structural basis for the increased affinities of IGF-1 and IGF-2 for isoform A of the insulin receptor is not understood completely, it is reasonable to suppose that the removal of the 12 amino acid residues from within the ligand binding pocket adjacent to binding Site 1 of the insulin receptor provides space to accommodate the C domain of IGF-1 or IGF-2. The difference in the relative affinities of the two isoforms for IGF-1 and IGF-2 may be due to differences between the C-domains of the two IGFs [103]. The C-domain is smaller in IGF-2 than in IGF-1 and may be accommodated more easily in the binding pocket of isoform B of the insulin receptor (Figure 5(a)).

### 3.7. Type I IGF Receptor

Type I IGF receptor and insulin receptor are thought to have evolved from an ancestral receptor involved probably in the regulation of both metabolism and growth. The sequences and overall structures of the two receptors remain very similar to the extent that they can form functioning heterodimeric hybrid receptors but the ligand specificity of and the signalling from the two receptors have diverged considerably. Of the three ligands, IGF-1 has the highest affinity, IGF-2 a slightly lower affinity and insulin has a much lower affinity (~100 fold) for type I IGF receptor [104] (Table 3).

As with the insulin receptor, each type I IGF receptor monomer comprises from the amino-terminus: the first leucine-rich repeat domain (L1), the cysteine-rich region (CR), the second leucine-rich repeat domain (L2), the first fibronectin type III domain (FnIII-1), the second fibronectin type III domain (FnIII-2), the third fibronectin type III domain (FnIII-3), the transmembrane domain, the juxtamembrane domain, the tyrosine kinase domain, and the C-terminal tail region. The insert domain of ~120 residues (ID) which contains the furin recognition site is within FnIII-2, and cleavage at this site yields the *α* and *β* chains of type I IGF receptor monomer.

Whereas the crystal structure of the entire ectodomain of the insulin receptor has been determined, the crystal structures of the type I IGF receptor are limited to the L1-CR-L2 domains [105] and intracellular tyrosine kinase domain [106]. Additionally, a solution structure of the ectodomain of the type I IGF receptor modelled on the insulin receptor structure has been determined by small-angle X-ray scattering [87]. Overall, the domain dispositions of the ectodomains of the type I IGF receptor are very similar to those of the insulin receptor. There are differences between the structures of the first three domains, L1-CR-L2, that may contribute to ligand specificity [107]. The first difference is in the structure of the L1 domain in the second and third *β*-strands of the second leucine-rich repeat. Phe39 in the second (central) *β*-sheet of the insulin receptor is involved in ligand binding and is a major determinant of the specificity of the receptor for insulin [108] whereas in type I IGF receptor, the equivalent residue Ser35 is not involved in ligand binding. The second difference is in the sixth module of the CR domain which is thought to govern IGF binding specificity. There are different amounts of a-helix and numbers of disulphide bonds in, and little sequence similarity between, the sixth modules of the two receptors [107]. Compared with the type I IGF receptor, the insulin receptor has a larger loop that protrudes into the ligand binding pocket (Figure 7(c)).

### 3.8. Interaction of IGF-1 and IGF-2 with Their Cognate Receptor

In contrast to insulin, IGF-1, and IGF-2 exist always as monomers in solution and have no dimer- or hexamer-forming surfaces. This difference raised the question of whether they interact with type I IGF receptor through two surfaces in a similar way to the interaction of insulin with the insulin receptor. The overall binding characteristics are similar to those of insulin. IGF-1 binding exhibits a curvilinear Scatchard plot indicative of the coexistence of more than one binding pocket, and binding shows negative cooperativity [109, 110]. The main difference is that unlike insulin, there is no loss of accelerated dissociation at high concentrations of IGF-1.

Identification of the binding surfaces on IGF-1 and IGF-2 has relied on mutagenesis and cross-linking studies (Figure 5(b)). Residues equivalent to insulin's Surface 1 together with residues in the C-domain of IGF-1 that are important for binding of IGF-1 to Site I of type I IGF receptor include Phe23, Tyr24, Tyr31, Arg36, Arg37, Val44, Tyr60, and Ala62 (Figure 5(a)). Alanine scanning mutagenesis has been useful in defining a second surface which may include Glu9, Asp12, Phe16, Leu54, and Glu58 and possibly the adjacent residues Ala8 and Met59 [111]. Thus, the binding of IGF-1 to type I IGF receptor is similar to that of insulin to the insulin receptor and involves many equivalent amino acids. Residues critical for the binding of IGF-2 to Site 1 of type I IGF receptor include Val14, Phe28, and Val43 and the second surface that engages potentially one or more of the FnIII domains is formed by Glu12, Phe19, Leu53, and Glu57 [112].

Analysis of the modelled solution structure of the ectodomain of type I IGF receptor reveals that at saturation concentrations of IGF-1, three molecules of the ligand are able to bind to the receptor. The asymmetry of the interactions led the authors to suggest that one ligand molecule binds with high affinity and bridges the two receptor monomers by interaction simultaneously with Site 1 on a first receptor monomer and Site 2 on the second receptor monomer. A further ligand molecule may bind to the available Site 2 on the first receptor monomer and a third ligand molecule to Site 1 on the second receptor monomer albeit at around 50-fold lower affinity. A small conformational change in type I IGF receptor ectodomain is required to allow it to bind three IGF-1 molecules simultaneously.

Following interaction of insulin, IGF-1 or IGF-2 with a receptor and its activation by autophosphorylation, the ligand receptor complexes are internalised. The activated ligand receptor complexes continue to signal in the intracellular vesicles. Eventually, the ligand dissociates within the endosomes, the receptors are inactivated by phosphotyrosine-specific phosphatases and recycled back to the plasma membrane, and the signal is attenuated [113]. As the pathways have been elucidated, it has become apparent that the same intracellular proteins transduce the signal from all three ligands and their different receptors. A major challenge now is to understand why different responses are manifest in different cells. The “cellular context” which is the sum of all the contributions from all the signal transduction molecules present in the cell is thought to be of great import [114].

### 3.9. Insulin and Type I IGF Hybrid Receptors

The discovery that the insulin receptor and type I IGF receptor could form heterodimeric hybrid receptors revealed an additional level of complexity [115–117]. Heterodimers form with the same efficiency as homodimers which means that the number of hybrid receptors is determined by the level of expression of each receptor and that if one receptor is present in excess of the other, there will be relatively few homodimers of the other receptor [118].

Early studies showed that the hybrid receptors have unusual binding properties; IGF-1 is displaced by low concentrations of IGF-1 but can only be displaced by high concentrations of insulin. In comparison, insulin is displaced by low concentrations of both insulin and IGF-1, with IGF-1 being more effective. These results suggest that IGF-1 inhibits allosterically insulin binding via interaction with type I IGF receptor monomer *α*-subunit in the hybrid receptor [117].

More recent analysis of the relative binding affinities and signalling capabilities of receptor heterodimers formed between either of the two insulin receptor isoforms and type I IGF receptor indicate that both hybrid receptors have a lower affinity for insulin than for either IGF-1 or IGF-2 (Table 3) [101, 119]. It has been suggested that the heterodimer between isoform A of the insulin receptor and type I IGF receptor may be considered as a relatively low specificity receptor with which IGF-1 and IGF-2 interact with high affinity to activate signal transduction. Although insulin has a lower affinity than the IGFs for this hybrid receptor, it could be activated by the high insulin concentrations present in insulin-resistant individuals with hyperinsulinemia in whom serum insulin concentrations can be as high as 0.8 nM [120, 121]. The high concentrations of therapeutic insulin required to reduce postprandial blood glucose in patients with diabetes mellitus type II reach 0.5 nM which is sufficiently high also to occupy this hybrid receptor [122].

### 3.10. Type II IGF Receptor and Insulin-Like Growth Factor Binding Proteins

Type II IGF receptor is located in the plasma membrane and interacts with IGF-1 and IGF-2. Type II IGF receptor has a higher affinity for IGF-2 than IGF-1 and does not bind insulin. It is also a receptor for mannose 6 phosphate for which it contains multiple interaction domains. Type II receptor does not transduce a signal but effectively lowers the bioavailability of IGF-1 and IGF-2 because after interaction, the ligand and receptor are internalised and degraded.

IGF-1 and IGF-2 are bound to insulin-like growth factor binding proteins (IGFBPs) in the circulation [123]. IGFBP3 is the major IGFBP with which IGF-1 and IGF-2 are complexed in the serum. IGFBPs protect IGF-1 and IGF-2 from degradation whilst in the circulation and deliver them to cells. The IGFBPs help to control the action of IGF-1 and IGF-2 by increasing their half-lives. The half-life can be increased from ten minutes to 15 hours when bound to IGFBPs. Because IGFs have a higher affinity for IGFBPs than for type I IGF receptor, the IGFBPs decrease the activation of the receptors and modulate the IGF response [124, 125].

## 4. Biological Effects and Consequences

### 4.1. Metabolic Effects of Insulin

Interaction of insulin with the insulin receptor initiates the recruitment of intracellular signal transduction molecules and culminates in the activation of metabolic pathways. Activation of the receptor causes tyrosine autophosphorylation on Tyr1146, Tyr1150, and Tyr1151 [126]. The receptor tyrosine kinase is now fully active and phosphorylation of juxtamembrane and carboxyl-terminal residues that form binding sites for docking proteins ensues. The sequence context of the phosphorylated residues determines which protein interaction domains are able to bind to the phosphorylated receptor and dictates thereby the docking proteins that are recruited and activated [127]. These adaptor molecules are in turn phosphorylated by the active receptor kinase. Effector proteins interact with phosphorylated adaptor proteins via different protein interaction domains such as SH3 and PDZ domains and the signal transduction cascade is initiated.

In man, the adaptor proteins are the insulin receptor substrates, IRS-1, IRS-2, and IRS-4, and Shc. The adaptor proteins have no intrinsic catalytic activity but transmit signal from the activated receptor to effector proteins. For example, IRS-1 interacts with the activated insulin receptor via a phosphotyrosine binding domain and with the inner side of the plasma membrane via its pleckstrin homology domain. IRS-1 is then phosphorylated on multiple tyrosine, serine, and threonine residues including two tyrosine residues within YXXM motifs, Tyr612 and Tyr632. Activation of phosphatidylinositol-3 kinase (PI-3 kinase) requires interaction of both its SH2 domains with these phosphorylated motifs [128].

**Figure 8:**
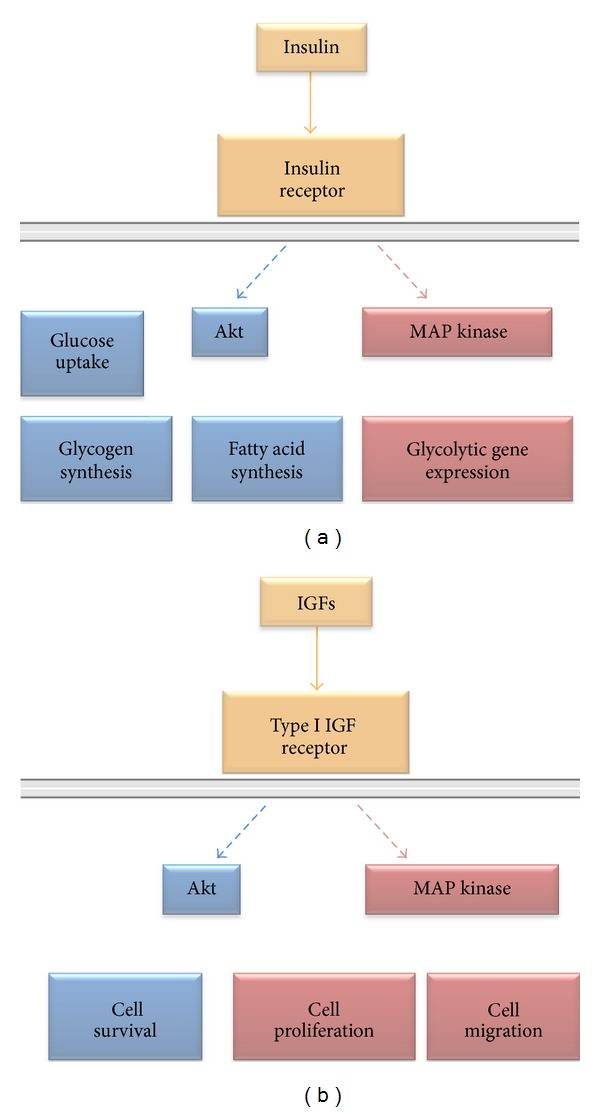
Graphic representation of the classic modes of insulin, IGF-1, and IGF-2 signal transduction. Insulin binds to the ectodomain of the insulin receptor and activates both the PI3-kinase and Akt pathway (blue) and the Ras and MAP kinase pathway (salmon pink). Activation of the Akt pathway results in translocation of GLUT4-positive intracellular vesicles to the plasma membrane and increased uptake of glucose, activation of glycogen synthase kinase 3 and the conversion of glucose to glycogen, and activation of pyruvate dehydrogenase and acetyl-CoA carboxylase and the conversion of glucose into the substrate for fatty acid synthesis. Activation of the MAP kinase pathway leads to increased expression of enzymes in the glycolytic pathway and preferential metabolism of glucose for energy. IGF-1 or IGF-2 (IGFs) bind to the ectodomain of type I IGF receptor and activate both the PI3-kinase and Akt pathway (blue) and the Ras and MAP kinase pathway (salmon pink). Activation of the Akt pathway results in inhibition of the Bcl-2 family of stress detection proteins and activation of GSK3*β*, both of which increase cell survival. Activation of the MAP kinase pathway leads to increased expression of genes that encode proteins important in regulation of cell cycle progression and hence to increased proliferation.

PI-3 kinase activation leads to phosphorylation of the protein kinase Akt on Thr308. Full activation of Akt requires a second phosphorylation on Ser473. Phosphorylation of Akt and hence its substrates is a key event in transmission of the insulin signal. Uptake of glucose into tissues that express GLUT4 glucose transporters, such as skeletal muscle and liver, is stimulated immediately after insulin is released into the blood stream. Insulin increases glucose transporter GLUT4 numbers at the cell surface by recruitment from an intracellular pool because activated Akt phosphorylates AS160 and TBC1D1 which are then phosphorylated by additional kinases. Phosphorylated AS160 and TBC1D1 activate small GTPases of the Rab family which are important in vesicle trafficking. GTP loading of Rab-GTPases in GLUT4-positive intracellular vesicles increases interaction with Rab effectors that control GLUT4-positive vesicle transport to the plasma membrane and hence uptake of glucose from the plasma (Figure 8).

Akt phosphorylates also glycogen synthase kinase 3 (GSK-3). GSK-3 is active only in its unphosphorylated form and is thereby inactivated. Its substrate, glycogen synthase is inactivated also by phosphorylation and therefore the inactivation of GSK-3 results in an increase in active, unphosphorylated glycogen synthase. This active glycogen synthase converts glucose into glycogen, the high-molecular-weight polymeric storage form of glucose. As well as generating a major energy source, conversion of glucose to a high-molecular-weight polymer solves elegantly the osmotic problems associated with high concentrations of a low-molecular-weight solute which are a major cause of tissue damage in diabetics (Figure 8).

Activated docking proteins stimulate also the transcription of insulin-regulated genes. For instance, the nucleotide exchange factor growth factor receptor-bound protein 2 (Grb2) binds the phosphorylated Tyr896 motif of IRS-1 via its SH2 domain [129]. Grb2 is then able to bind the guanine nucleotide exchange factor, son of sevenless (SOS), which in turn catalyses the replacement of bound GDP with GTP on the Ras GTPase. This protein phosphorylation cascade culminates in activation of mitogen-activated protein kinases (MAPKs), which enter the nucleus and phosphorylate nuclear transcription factors. Effects of insulin on glycolysis are mediated by increased expression of hexokinase, phosphofructokinase, and pyruvate kinase, the enzymes that control the three irreversible steps of glycolysis (Figure 8).

Insulin can stimulate nonmetabolic effects via interaction with the insulin receptor and activation of the MAPK pathway [130]. This role of insulin may be more significant in cancer cell biology than recognised previously. There is evidence that the mitogenic response to insulin is more pronounced after activation of isoform A of the insulin receptor, which is overexpressed in some tumour cells, than after activation of isoform B of the receptor [104]. In cancer patients with insulin resistance, the consequent high levels of circulating insulin, together with overexpression of the insulin receptor by the tumour cells, stimulate significant nonmetabolic effects [100].

### 4.2. Biological Actions of IGF-1 and IGF-2

As for the insulin receptor, activation of type I IGF receptor occurs after ligand interaction in the extracellular domain. This interaction induces small conformational changes which lead to an increase in the intrinsic kinase activity of the receptor and autophosphorylation of Tyr1131, Tyr1135, and Tyr1136 residues in the kinase domain [131].

The classic function of both IGF-1 and IGF-2 is to stimulate growth. The major signal transduction pathways responsible is thought to be the Ras and MAPK pathway (Figure 8). After phosphorylation of IRSs or Shc, the Grb2 protein is recruited via its SH2 domain. Grb2 and SOS are then able to interact via an SH3 domain which brings SOS in close proximity to the membrane anchored Ras and reverses its autoinhibition. SOS promotes dissociation of GDP from Ras which allows GTP to bind, and after dissociation from SOS, active GTP-bound Ras phosphorylates its substrate Raf and the signal cascade is committed. Ultimately, the MAP kinase enters the nucleus to alter the expression of its target genes, and cells are driven through the cell cycle.

Activation of the protein kinase Akt by phosphorylation via the same pathway as described above for insulin is considered important in the prosurvival effect of IGFs (Figure 8). Activated Akt promotes cell survival via multiple effectors. One important pathway involves the Bcl-2 family of proteins. Akt phosphorylates BAD a proapoptotic member of the “stress detecting” Bcl-2 family of proteins. BAD induces apoptosis by interaction with and inhibition of the antiapoptotic Bcl-2 family members Bcl-xL and Bcl-2, which displaces them from interaction with proapoptotic proteins such as Bak and Bax. The liberated Bak and Bax are then able to aggregate and increase the porosity of mitochondria. The mitochondria release cytochrome c which activates caspases and induces caspase-dependent cell death. Because phosphorylated BAD does not form heterodimers with Bcl-xL or Bcl-2, phosphorylation of BAD by Akt inactivates BAD and allows proteins like Bcl-2 to inhibit proapoptotic proteins. Akt activates also by phosphorylation GSK3*β*. GSK3*β*-dependent cell survival implicates nuclear factor-*κ*B activation [132] and negative regulation of both intrinsic and extrinsic caspase-dependent proapoptotic pathways [133].

IGFs may also be involved in angiogenesis through induction of HIF-1*α* and VEGF-C and by direct actions on vascular and lymphatic endothelial cells [134–139]. The effects of IGFs on invasion and metastasis include disruption of *β*-catenin and E-cadherin complexes which promotes cell detachment. IGFs promote invasion by regulating the expression of matrix metalloproteinases (MMPs) and urokinase plasminogen activator receptor system, both of which are involved in degradation of the extracellular matrix (ECM) in cancer [140, 141].

### 4.3. Differential Insulin, IGF-1, and IGF-2 Signal Transduction from Isoform A of the Insulin Receptor

There is evidence for subtle differences in insulin and IGF-2 signal transduction via isoform A compared to via isoform B of the insulin receptor; insulin stimulates mainly glucose uptake and IGF-2 stimulates preferentially proliferation [142]. Signals through the A isoform may recruit a different repertoire of proteins from signals through to the B isoform [143] and may deliver mitogenic signals in addition to metabolic signals [144–146].

Although the molecular basis of the differences in signal transduction is not understood completely, it may result from either or both of two properties: the residency time of the ligand on the insulin receptor and the ratio of affinities for the insulin and type I IGF receptors. Glu12 in IGF-2 and its equivalent Glu9 in IGF-1 are located on binding Surface 2 of the ligands and are important for high-affinity binding to the A isoform of the insulin receptor and type I IGF receptor. Introduction of a positive charge at this location in IGF-2 reduces affinity for both receptors and introduction of a positive charge at the equivalent position in IGF-1, Glu9Lys, reduces affinity for type I IGF receptor [147]. In insulin, the equivalent residue is HisB10 and substitution with an aspartic acid or glutamic acid acidic residue creates an analogue with a reduced “off-rate” from the insulin receptor and increased affinity for and activation of type I IGF receptor. This analogue has increased mitogenicity and increases mammary tumourigenesis in rats, supporting the contention that the kinetics of interaction of a ligand with each receptor contribute to the final biological effect stimulated by the interaction [148, 149].

### 4.4. Role of Insulin, IGF-1, and IGF-2 in Cancer

There is little evidence for the dramatic overexpression or mutation of components of the insulin and IGF signal transduction pathways in malignant cells, or for corruption by transforming-viruses, that led to the identification of other oncogenes such as epidermal growth factor receptor or ErbB-2. The absence of oncogenic evidence for the importance of the IGF system in cancer delayed the realisation of its import [150–154]. There is however compelling evidence, included from animal models of tumour formation and progression, for the importance in IGFs in carcinogenesis. The question in the context of the present review is the extent to which the insulin and IGF signal transduction system is responsible for the effects of obesity and diabetes on cancer risk and progression.

### 4.5. Insulin and the Insulin Receptor Isoforms and Cancer Risk

The possibility that the hyperinsulinaemia in individuals who develop metabolic syndrome predisposes towards cancer implicates a direct effect of high plasma insulin concentrations. There is evidence that plasma levels of insulin or the C-peptide, which is the peptide that is cleaved from insulin during its biosynthesis, are associated with colon [155–158], endometrial [159], breast [158, 160], prostate [161] and pancreatic [162] cancers. Similarly, the hyperinsulinaemia that occurs during treatments for diabetes could be causative.

Epidemiological studies of cancer risk in diabetic patients treated with the biguanide, metformin provide support for the importance of hyperinsulinaemia in diabetes-associated cancer risk. Diabetic patients who do not take metformin to control their diabetes have a 40% higher cancer burden compared to diabetic patients who do [23, 163]. Metformin reduces the levels of circulating insulin and decreases the levels of glucose in the blood; it is probably through these effects that metformin decreases cancer risk.

In 1990, it was found that insulin receptors are often expressed at higher levels on tumour cells than on classic insulin target cells [164]. This high expression was unexpected as the majority of cancers do not derive from the principle target organs of insulin, and cancer cells have highly effective insulin-independent glucose uptake mechanisms [165]. It is now recognised that the insulin receptor is expressed by many malignant tumours [100] including those of prostate [95], lung [166], thyroid, colon [92], and breast [164]. Studies have identified transcription factors and cofactors that regulate expression of insulin receptor in tumour cells [167–169].

The isoform of the insulin receptor present may be important in the development of cancer. The major insulin receptor isoform expressed on cancer cells is the A isoform which has a higher affinity for insulin than the B isoform and is more readily activated by circulating insulin. Isoform A may also provide a more mitogenic stimulus than the B isoform which would be associated with a higher cancer risk [170]. Another mechanism by which insulin could increase the risk of cancer in obese individuals with hyperinsulinaemia is by direct stimulation of type I IGF receptor. Although the affinity of insulin for type I IGF receptor is low, the supraphysiological concentrations of insulin present in obese people with insulin resistance and hyperinsulinaemia and in those with the metabolic syndrome are probably sufficiently high to signal through this receptor. Serum concentrations of insulin can reach 0.8 nM, at which concentration insulin would occupy and activate type I IGF receptor [120, 121] (Table 3).

The hyperinsulinaemia that occurs in obesity may increase cancer risk by indirect mechanisms. Hyperinsulinaemia lowers the plasma concentrations of IGFBP-1 and IGFBP-3 [171, 172] which increases free circulating IGF-1. Chronic hyperglycaemia decreases IGFBP-2 to increase bioactive IGF levels [173]. High insulin concentrations in the hepatic portal circulation increase growth hormone receptor expression, thereby augmenting IGF-1 production [174]. In essence, obese individuals with hyperinsulinaemia and hyperglycemia will have higher concentrations of free circulating IGF-1 and augmented activation of the IGF-dependent proliferation pathway.

Therapeutic insulin has been shown to increase cancer risk in numerous studies. Further, it has been shown that the duration of insulin treatment is associated positively with cancer incidence [175]. More recent studies have investigated if treatment with insulin analogues is associated with a similar or greater risk than treatment with unmodified insulin. Treatment of diabetes mellitus type 2 patients with the long-acting insulin analogue, glargine, was found to be associated with an increased incidence of all cancers compared to natural therapeutic insulin in two studies [176, 177] and in pancreatic and colorectal cancer in a third study [178] whereas in a fourth study the increased risk for breast cancer was equal to unmodified insulin [179]. These conclusions were controversial and provoked extensive debate because of the implications for diabetics and pharmaceutical companies [149]. Reservations about the methodology and comparison groups have been expressed and larger studies are underway. One recent study confirmed an increased risk of breast cancer for patients given glargine, compared with those given natural insulin [180].

### 4.6. Insulin and the Insulin Receptor Isoforms and Cancer Progression

There has been a steady increase in the interest in the importance of insulin and the insulin receptor in cancer progression. High fasting insulin levels are associated with higher risk of colorectal adenoma recurrence [181] and with a poor breast cancer prognosis [182]. High serum insulin C-peptide levels are associated with a worse prognosis in prostate [26] and breast cancers [183]. More rapid tumour progression is associated with hyperinsulinaemia, and the dependence has been confirmed in animal models [184].

The type of insulin receptor isoform present in malignant cells may play a role in the progression of cancer as well as in cancer risk. Studies on breast cancer have shown that the ratio of isoform A to isoform B is important in determining the nature of the cancer [185]. Women with an increased ratio of isoform A to isoform B have more aggressive and rapidly progressive breast cancer [186]. The ability of the insulin receptor to form hybrids with type I IGF receptor may contribute to the effects of insulin on cancer progression. In essence, the impact of insulin on cancer risk may be large because of its capacity to deliver a more mitogenic, IGF-like signal either through isoform A of the insulin receptor or through insulin receptor: type I IGF receptor hybrids.

### 4.7. IGFs and Cancer Risk

Meta-analyses and recent large studies on the relationship between height and cancer risk have shown that height is a risk factor for some common cancers. The most consistent and convincing associations are for colorectal, breast, and prostate cancers [187]. Interpretation of the epidemiological data is that increased height reflects higher circulating levels of the growth stimulatory hormones particularly the IGFs during prepubertal development. The length of the long bones in the legs is a major contributor to adult height and is dependant largely on exposure to IGFs which is partly determined by genetics and partly by prenatal and childhood nutrition [188].

More direct evidence for a role for IGFs in the development of cancer was provided in 1998 when Hankinson et al. reported that the risk of breast cancer was associated with the levels of circulating IGF-1 and IGFBP-3 [189]. Notably, there was a large increased relative risk of 7.3 for developing breast cancer in premenopausal women who were in the top tertile for high serum IGF-1 concentrations and had low circulating IGFBP3 concentrations. A high ratio of IGF-1 to IGFBP3 means that more IGF-1 is bioavailable. Similar findings were reported for prostate cancer risk [190] with a 2.4-fold higher risk for men in the highest quartile of IGF-1 concentration compared to men in the lowest. The following year, an association was reported for colorectal cancer [191]. In 1999, a small increased risk of lung cancer was reported in people with elevated serum IGF-1 [192].

Thus, by the turn of the century, the prevailing view corroborated by clear evidence was that there is increased risk of common cancers in people with elevated circulating IGFs and low circulating IGFBPs, which supports earlier conclusions based on the association between height and cancer risk [193].

### 4.8. IGFs and Cancer Progression

Normal levels of circulating IGFs are sufficient to stimulate type I IGF receptor but tumour stroma and malignant cells can produce higher intratumour concentrations of IGFs. There are few reports of autocrine IGF-1 production but reports of autocrine IGF-2 production are common. IGF-1 is expressed in colon carcinoma [194, 195] and IGF-2 is expressed in breast [196, 197], prostate [198], colorectal [199], adrenocortical [200] liver [201, 202], and thyroid [94] carcinoma cells and several types of sarcoma including osteosarcomas, myosarcomas, fibrosarcomas, and Ewing's sarcoma [203, 204]. Such high intratumour IGF concentrations enable heightened IGF signal transduction and consequent cancer cell proliferation, survival, and invasion.

Most studies on cancer progression have focussed on type I IGF receptor and the intracellular IGF signal transduction pathway. Type I IGF receptor and components of the IGF signal transduction pathways are expressed by most tumour types [152, 154, 205–207]. In some cancers, increased type I IGF receptor expression is associated with worse disease prognosis. In colorectal cancer, there is a stepwise increase in the expression of type I IGF receptor during progression from colonic adenomas towards primary colorectal adenocarcinomas and metastases [208]. For breast cancer, one study found that higher levels of type I IGF receptor were correlated with a worse prognosis for all tumours and in oestrogen receptor-negative tumours [209] whereas another [210] found no prognostic value. In prostate cancer, there is a significant increase in type I IGF receptor mRNA and protein expression in primary tumours and in bone metastases, compared to benign prostatic epithelium [211, 212]. Similar associations have been reported for less common cancers including synovial sarcoma [213], melanoma [214], and gastric [215] and renal clear cell carcinomas [216]. Some studies have shown that high levels of receptor or phosphorylated receptor are associated with a poor prognosis [166, 217].

IGFs help metastatic cells adapt to a new environment although the mechanisms are not well defined. IGF-1 facilitates the establishment of both lung [218] and colon [219] cancer metastases in the liver. IGFs may be involved also in metastasis of prostate cancer to bone. Osteoblasts and bone endothelial cells produce IGFs and IGFBPs [220, 221] whilst prostate cancer cells produce uPA [222] which degrades IGFBPs and hence increases the bioavailability of IGFs [223].

### 4.9. Cooperation between Obesity-Associated Factors in Cancer Risk

Obesity aggregates several risk factors, some of which are particularly relevant to certain types of cancer. For example, hyperinsulinaemia, diabetes, and serum IGF levels are all independent risk factors for breast and colon cancers and may account for the increased risk associated with obesity. Hyperinsulinaemia and diabetes are associated with endometrial cancer. The associations identified may reflect the scope of the different epidemiological studies or they may reflect divergent cancer cell biology. There are clear interactions between obesity, insulin, and IGFs which compound their effects on cancer risk. In addition, steroid hormones regulate the expression of components of the IGF signal transduction pathway in some cancer cells and as a consequence can increase the cancer risk associated with hyperinsulinaemia, diabetes, and high serum IGF levels.

Hyperinsulinaemia stimulates androgen synthesis in the ovary, increases aromatisation of androgens to oestrogens, and decreases hepatic production of sex hormone-binding globulin which leads to increased bioavailable androgens and oestrogens [224, 225]. As circulating concentrations of insulin and IGF-1 rise with increasing obesity, the levels of the sex hormone-binding globulins decrease [226]. For instance, a woman with a BMI of >30 kg/m^2^ has half as much sex hormone-binding globulin as a woman with a BMI of <22 kg/m^2^ [227]. In normal or premalignant cells, the higher concentrations of, for example, bioavailable oestrogens present in obese women could augment the proliferative and anti-apoptotic effects of insulin and the IGFs with potentially oncogenic consequences.

There is some evidence that insulin resistance and high levels of IGFs, which are associated with central obesity, may play a more important role in premenopausal breast cancer while oestrogen may play a greater role in postmenopausal breast cancer [189, 228]. Furthermore, obesity influences the type of tumour that arises. For example, basal-like or triple-negative breast tumours which do not express oestrogen and progesterone receptors or HER2 are more prevalent in obese women [229, 230]. Interestingly, cell proliferation and survival of triple-negative breast cancer cells is IGF dependent [231].

### 4.10. Cooperation between Obesity-Associated Factors in Cancer Progression

The interaction between steroid hormones and the IGF signal transduction pathway plays an important role in cancer progression and has been studied mainly in breast cancer. The significance of oestrogens in progression *in vivo *was demonstrated by Beatson [232] but it was not clear if there was a direct effect on breast epithelial cells. This question was resolved when cultured malignant breast cells were shown to contain oestrogen receptors [233] and their proliferation was shown to be oestrogen responsive [234, 235]. It became apparent subsequently that oestrogens control proliferation by modulating the effect of IGFs rather than by regulating the expression of genes involved in the cell cycle. The synergistic effects of oestrogens and IGFs on proliferation [78, 236] suggested that oestrogens might control the expression of components of the IGF signal transduction pathway. There is evidence that oestrogen increases expression of IGF-2 [237] but there is most likely an adequate supply of IGFs from the circulation and the stroma *in vivo* and in tissue culture medium *in vitro*. The main way that oestrogens potentiate the response of breast cancer cells to IGFs is probably by induction of the expression of components of the IGF signal transduction pathway [238] including type I IGF receptor [78, 239], IRS-1 [240–242], and IRS-2 [243]. Oestrogens may also regulate IGF-stimulated cell migration via increased IRS-1 and IRS-2 expressions as these are both involved in breast cancer cell migration [243].

Despite the association between IGFs and cancer progression and the androgen responsiveness of prostate cancer, there is little evidence of interaction between androgens and IGFs in an analogous way to oestrogens and IGFs in breast cancer. Androgens increase type I IGF receptor expression in prostate cancer cells [244] but few studies have investigated if androgens and the IGF signal transduction system interact to stimulate prostate cancer cell growth.

## 5. Treatment Targeted to Adiposity-Induced Cancers

Cancers that have arisen in obese or diabetic individuals will have identifiable properties that will allow them to be treated with specific therapies. These properties will reflect the forces that drove their origin and progression. We have presented evidence in this review that cancers that arise in a background of obesity and diabetes are dependent on insulin, IGFs, and their interactions with steroid hormones. Breast cancer is treated commonly with oestrogen-based endocrine therapies. More recently, the appreciation that some cancers are responsive to IGFs has led to the development of drugs that target the IGF signal transduction pathway and in some instances the insulin signal transduction pathway.

Strategies for targeting the IGF signal transduction system include antibodies to the cell surface receptors, tyrosine kinase inhibitors, siRNA [245], dominant negative constructs [246, 247], antibodies to the ligands [235], and IGF binding proteins. 

### 5.1. Antibodies to Type I IGF Receptor

**Figure 9:**
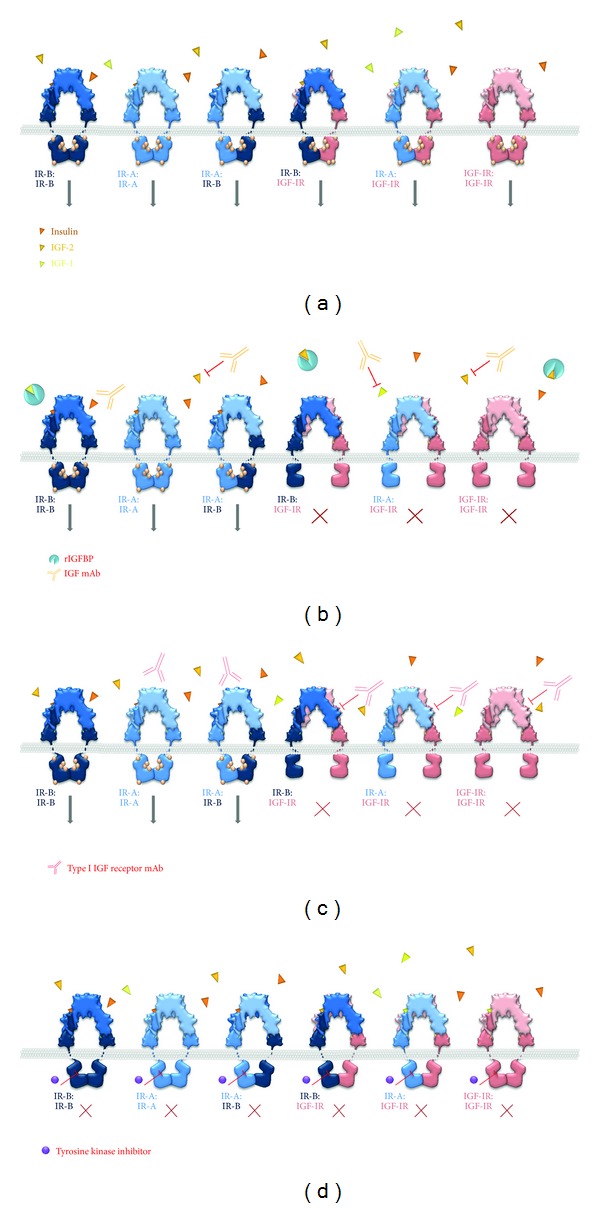
Therapeutic strategies to inhibit insulin, IGF-1, and IGF-2 signal transduction. Graphic representations of the insulin receptor isoform B (IR-B, dark blue), the insulin receptor isoform A (IR-A, lighter blue), and of the type I IGF receptor (IGF-IR, pink) are shown as homodimeric or heterodimeric hybrid receptors. For each receptor monomer, the *α*-chain is coloured slightly lighter than the *β*-chain. Bars indicate the approximate positions of the single disulphide bonds between the C647 in the *α*-chain and C860 in the *β*-chain of each receptor monomer (yellow); the single disulphide bond between the FnIII-1 domains of each of the two *α* chains in each dimer is obscured. The extracellular ligands, insulin (orange), IGF-2 (ochre), and IGF-1 (pale yellow), are shown as triangles. The downward pointing grey arrows indicate that a signal has been transduced from a receptor dimer (a). The deep yellow spheres attached to the ectodomains of the activated receptors represent phosphorylated residues. Antibodies against IGF-1 and IGF-2 (IGF mAb; yellow), and recombinant insulin-like binding protein as an incomplete sphere (rIGFBP; light blue), are shown. Inhibition of a signal transduction molecule that is affected by one of the inhibitors is indicated by an inhibition symbol (brick red). Absence of a signal from a receptor dimer is indicated by a red cross below the receptor dimer (b). Monoclonal antibodies against IGF-1 and IGF-2 will not inhibit insulin signal transduction. Recombinant IGFBP will inhibit IGF-1 and IGF-2 but will not inhibit insulin. Antibodies specific for type I IGF receptor are shown (Type I IGF receptor mAb; deep pink) (c). Antibodies specific for the type I IGF receptor will inhibit type I IGF receptor homodimer and its hybrid receptors formed with both isoforms of the insulin receptor but will not inhibit insulin receptor homodimers or hybrids of the two isoforms of the insulin receptor. Representations of tyrosine kinase inhibitors (TKIs) that inhibit both the insulin and type I IGF receptors (tyrosine kinase inhibitor; violet) are illustrated (d). Tyrosine kinase inhibitors that affect type I IGF receptor and the insulin receptor will inhibit signal transduction via all the insulin and IGF signal transduction pathways. Tyrosine kinase inhibitors that are specific for the type I IGF receptor will inhibit the type I IGF receptor homodimer and its hybrid receptors but will not inhibit insulin receptor homodimers or hybrids of the two isoforms.

The component of the IGF signal pathway against which most potential drugs have been developed is type I IGF receptor. A number of humanised monoclonal antibodies specific for type I IGF receptor have been produced including figitumumab, cixutumumab, ganitumab, dalotuzumab, AVE1642, and R1507 [248–253]. They are of different subclasses but are generally long lasting with a half-life of greater than 10 days. After interaction with the ectodomain of the receptor, they promote receptor internalisation and lysosome-mediated receptor degradation. Although the antibodies do not recognise directly insulin receptors, they can bind to and inhibit the activity of hybrid receptors that are formed with either the A or B isoform of the insulin receptor (Figure 9) [153, 254]. The antibodies should have therefore activity in cells that express insulin receptors as long as type I IGF receptor is in excess.

Type I IGF receptor targeted antibodies have some undesirable side effects, notably hyperglycaemia. Blockade of type I IGF receptor in the hypothalamic pituitary axis is interpreted as a fall in circulating IGF-1 and as a consequence, growth hormone secretion is elevated. The net effect is a twofold increase in serum growth hormone concentrations, a modest increase in fasting glucose (<20%) and an elevation in fasting insulin [248]. The induction of hyperglycaemia is thought to result from peripheral insulin-resistance associated with elevated levels of growth hormone. This side effect has resulted in the inclusion of growth hormone antagonists and glucose-lowering agents in some clinical trials.

There are upwards of 50 ongoing clinical trials of type I IGF receptor antibodies in combination with other treatments such as radiotherapy, chemotherapy, or inhibitors of other signal transduction pathways. Trials of receptor antibodies in combination with irradiation and chemotherapeutic drugs are based on the observations that type I IGF receptor causes radio- and chemoresistance in cell culture [255, 256] and in xenograft models by reducing apoptosis and increasing DNA replication and the repair of double-stranded breaks [257–261]. Trials have yielded mixed results with some being abandoned because of the frequency of adverse events or the low probability of achieving the endpoint of increased survival. Responses to single-agent therapy have been observed in sarcomas and adrenocortical carcinoma; prolonged periods of stable disease have been reported [251, 253, 262, 263]. Some regard these results as disappointing but others regard them as encouraging because the trials have been on unselected, heavily pretreated patients [264].

### 5.2. Small Molecule Inhibitors of Tyrosine Kinase Activity

The majority of the small molecule inhibitors that have been developed compete with ATP to bind to the catalytic tyrosine kinase domain of type I IGF receptor and hence inhibit its enzymatic activity: linisitinib, BMS-754807, and INSM-18 [265, 266]. An exception is the picropodophyllin, AXL-1717, which is a non-ATP competitive inhibitor of the receptor kinase. The small molecule inhibitors are characterised by a short half-life which allows for easier control of their activity but requires frequent administration. They have the advantage compared with the therapeutic antibodies that they may be administered orally and may cross the blood brain barrier. They are generally less specific for type I IGF receptor than the antibodies, but have varying degrees of specificity for type I IGF receptor (Figure 9). For example, AXL-1717 is highly selective for type I IGF receptor whereas linisitinib inhibits the tyrosine kinases of both the insulin and type I IGF receptors. BMS-754807, is predominantly a dual type I IGF receptor and insulin receptor inhibitor which inhibits MET, TRK A, TRK B, Aurora A, and Aurora B kinases at high concentrations and INSM-18 inhibits type I IGF and insulin receptors and ErbB2 [254, 267].

Clinical trials of the tyrosine kinase inhibitors are not as advanced as trials of the inhibitory antibodies. The former are well tolerated and have shown antitumour activity in phase I dose escalation trials. They are being evaluated currently as single agents for the treatment of cancers in which the early trials showed evidence of a response, or in combination with cytotoxic drugs or inhibitors of other signal transduction pathways [264, 265].

### 5.3. Alternative Targeting Strategies

Therapeutic antibodies to the IGFs, and recombinant IGF binding proteins, are being developed to inhibit the effects of IGFs. MEDI-573 is a humanised monoclonal antibody that interacts with both IGF-1 and IGF-2. MEDI-573 prevents the IGFs binding to type I IGF and insulin receptors without affecting glucose homeostasis [268]. Cell growth of implanted malignant prostate cells is arrested in an animal model [269] and MEDI-573 has stabilised disease progression in phase I trials [254]. Recombinant IGFBP3 has been shown to lower the bioavailability of IGFs and to reduce tumour growth rate in animal models of trastuzumab-resistant breast cancer and of lung and colon cancers [270, 271].

### 5.4. Biomarkers of IGF Responsiveness

Stratification of patients on the basis of biomarker measurements has been shown to identify patients who are most likely to benefit from particular forms of therapy. For example, breast cancer patients are selected for hormone therapy based on oestrogen receptor or progesterone receptor status [272], and trastuzumab therapy is based on ErbB2 expression [273]. In colorectal cancer, epidermal growth factor receptor inhibitors such as cetuximab are of benefit only in patients with a nonmutated *KRAS* gene [274, 275].

Identification of patients who would benefit from inhibitors of the IGF signal transduction pathway is at an early stage. Studies with figitumumab have shown greater benefit in patients with high compared to those with low free IGF-1 levels in both non-small-cell lung carcinoma [276] and sarcoma [263] and a trend towards improved response in those with higher tumour type I IGF expression in non-small-cell lung carcinoma [254]. Other studies have suggested that expression of components of the IGF signal transduction pathway may help to predict responsiveness to a type I IGF receptor antibody [277] or have focussed on expression of IRS-1 or of the A isoform of the insulin receptor [204, 278]. Another approach may be to use molecular profiling to identify an IGF-responsive signature [279]. Alternatively, greater response to figitumumab was obtained in non-small-cell lung carcinomas with squamous histology which indicates that some tumour subtypes may be more responsive than others [254].

### 5.5. Inhibition of the Insulin Signal Transduction System

As described above, the high degree of similarity between the autophosphorylation catalytic domain of type I IGF and insulin receptors has meant that the majority of tyrosine kinase inhibitors interact with the two receptors with equivalent affinity (Figure 9). The realisation that the A isoform of the insulin receptor is expressed at high levels in many tumour cells has altered perceptions of this dual specificity which is viewed now by many as being advantageous.

Preclinical evidence indicates that the insulin receptor may compensate for the therapeutic inactivation of type I IGF receptor. Insulin receptor expression is increased in type I IGF receptor-null mice which allows normal growth of the animals [280] and the insulin receptor promotes resistance to inhibitory type I IGF receptor antibodies in a transgenic pancreatic and a mouse mammary tumour animal model [267, 281]. Thus, an unforeseen benefit of the inhibitors that do not discriminate between the two receptors may be a superior clinical response.

It was expected that inhibition of the insulin receptor would lead to toxic levels of hyperglycaemia given the importance of the insulin receptor in the regulation of blood glucose. However, a recent study showed that administration of the tyrosine kinase inhibitor, BMS-536924, did not induce significant hyperglycaemia as it does not accumulate in muscle at levels sufficient to block insulin-stimulated glucose uptake [282].

Metformin, the biguanide taken by diabetes mellitus type 2 patients to lower their blood glucose levels, protects them against cancer. The exact protective mechanism is unknown and there is no evidence to suggest that metformin would be protective in nondiabetics. Nonetheless, data are encouraging and suggest that further research into biguanides and their role in cancer protection is warranted [283]. There is evidence from studies* in vitro *that metformin may have direct antitumour activity on tumour cells [284–287]. The demonstration that thiazolidinediones are peroxisome proliferator-activated receptor-*γ* (PPAR*γ*) agonists suggests that they may have antiproliferative activity via repression of insulin receptor expression [288].

### 5.6. Steroid Hormone-Responsive Cancer

A few cancers are hormonally responsive of which the two most common are breast and prostate. Oestrogen and androgen dependence were identified as therapeutic targets for the treatment of breast and prostate cancers by oophorectomy and orchidectomy, respectively, before the molecular details of their mechanisms of action were known [232, 289]. A significant proportion of breast and prostate cancers are responsive to oestrogens and androgens, respectively; endometrial and ovarian cancers can respond to oestrogens and leukaemia responds to glucocorticoids. Current treatment targets either the supply of steroid hormone to the tumour cells or the interaction of the steroid hormone with its receptor.

In the context of the present review, the importance of endocrine therapy is in its value in combination with insulin- or IGF-targeted therapeutic strategies. The rationale is that because steroid hormones sensitise cancer cells to the tumour-promoting actions of insulin and IGFs, simultaneous targeting will potentiate the insulin- and IGF-suppressant effects. Present evidence indicates that this therapeutic approach is most likely to be effective in breast cancer but may have value also in endometrial and ovarian cancers [290–294].

### 5.7. Inhibition of Steroid Synthesis

There are several different modalities of treatment designed to reduce oestrogen production. These therapies include surgical, cytotoxic chemical- or radiotherapeutic suppression of ovarian function, or inhibition of pituitary stimulation of ovarian function in pre- or perimenopausal women. Oestrogen production may be prevented by enzyme inhibition: 17 *α*-hydroxylase inhibitors such as abiraterone and orteronel prevent conversion of pregnenolone and progesterone into androstenedione, 5 *α*-reductase inhibitors such as finasteride and dutasteride prevent reduction of testosterone to the more potent dihydrotestosterone, and aromatase inhibitors such as exemestane and anastrozole prevent aromatisation of testosterone into oestradiol and of androstenedione into oestrone (Figure 4(b)).

In males and postmenopausal females, adipose tissue provides the major source of the enzymes 17*β*-hydroxysteroid dehydrogenase, which converts androstenedione into testosterone, and aromatase which converts androgens into oestrogens. It seems logical to suggest that the above therapies which target steroid synthesis might be particularly effective in the obese and many diabetic individuals who have abundant reservoirs of adipose tissue to produce the enzymes involved in steroid production.

### 5.8. Competitive Inhibitors of Binding of Steroids to Their Receptors

Steroid hormones are lipid-soluble and diffuse across the plasma membrane to interact with intracellular or intranuclear receptors. Responsiveness to steroid hormones is determined by the presence of steroid hormone receptors in the target cells of which there is at least one cognate receptor protein for each type of steroid hormones (Figure 4(a)). The steroid hormones bind to and activate their cognate receptors which are ligand-dependent transcription factors and thereby alter directly the expression of repertoires of hormone-responsive genes.

Antioestrogens are competitive inhibitors of oestrogen for the oestrogen receptor (Figure 4(c)). Competitive inhibitors can be partial oestrogen antagonists that do not stimulate some oestrogenic effects but do stimulate other oestrogen effects. The partial antagonist tamoxifen has been the antioestrogen used most widely. Tamoxifen is a triphenylethylene derivative that binds to the oestrogen receptor but does not induce the conformational change in the position of helix 12 relative to the ligand binding domain that is required for subsequent interaction with coactivators of transcription [295]. Tamoxifen and its metabolites have both oestrogenic and antioestrogenic activities in breast cancer cells [296, 297]. Pure oestrogen antagonists such as the steroidal antioestrogen, fulvestrant, have no oestrogen agonist activity [298]. Antiandrogens or androgen antagonists are the mainstay of systemic treatment of prostate cancer and may have a role in the treatment of some epithelial ovarian cancers. Flutamide and bicalutamide are widely used pure antiandrogens.

### 5.9. Predictive Biomarkers of Responsiveness to Hormone Therapy

Accepted predictive biomarkers of response to oestrogen-targeted therapy are the oestrogen and progesterone receptors. Other oestrogen-responsive gene products such as TFF1 have potential [299, 300]. In the context of combination therapy with insulin- or IGF-targeted drugs, measurement of important proteins in the IGF signal transduction pathway that are known to be induced by oestrogens would be apposite. Type I IGF receptor, IRS-1, IRS-2, and IGF-2 have all been shown to be induced by oestrogen in breast, and type I IGF receptor in endometrial and ovarian, cancer cells, and should be investigated as biomarkers for combination therapies.

## 6. Conclusions

Great strides have been made in understanding the pathophysiology that underlies the association between obesity and cancer risk. We have discussed the important contributions of hyperinsulinaemia, diabetes, exogenous insulins, and insulin-like growth factors and the influence of steroid hormones. The evidence for the relatively greater importance of visceral than subcutaneous obesity and of local or paracrine effects of IGFs and steroid hormones secreted by adipose tissue is overwhelming. Surgical intervention is effective and drugs to fight obesity by altered appetite or metabolism are available albeit with significant side effects. As obesity is preventable and reversible, the most effective cancer prevention strategy should be successful encouragement of a healthy lifestyle to control weight.

The effects of obesity and diabetes on cancer progression are mediated by the same pathways as cancer risk. This review has focused on insulin, insulin-like growth factors, and how their effects may be enhanced by steroid hormones. Conventional therapies have been used to target steroid hormone action for many years, and new therapies are being evaluated. Novel drugs that target the IGF signal transduction pathway, and in some cases the insulin signal transduction pathway in addition, have been developed and are in clinical evaluation. It is opportune that the availability of drugs which are predicted to be effective in cancers that arise in a background of obesity, hyperinsulinaemia, and diabetes has coincided with the onset of the obesity-associated cancer epidemic.

It is fortuitous also that the onset of epic morbidity induced by altered hormonal balance and homeostasis disruption has been paralleled by enormous advances in our appreciation of the molecular mechanisms that underlie the interactions of insulin and IGFs with their receptors and their subsequent actions. Formerly, the distinctions between insulin, IGF-1, and IGF-2 and the biological effects that they stimulated via their cognate receptors were clear. We appreciate now that there is considerable redundancy and cross-interaction and that the same signal transduction proteins are activated by all three ligands. In malignant cells, high local IGF-2 concentrations, isoform A of the insulin receptor and hybrid receptors contribute to the significant insulin- and IGF-dependency. The ability of the malignant cells to respond to endogenous hyperinsulinaemia or to therapeutically induced hyperinsulinaemia or to the high local concentrations of IGF-1 and IGF-2 secreted from visceral adipose tissue in the case of endometrial and oesophageal cells or from mammary adipose tissue in the case of breast cells will be influenced by increased expression of isoform A of the insulin receptor and the type I IGF receptor. The biological response to the ligand is determined by the target cell as much or more than by which of the three ligands interacting with which receptor. The response of the target cell is dictated by the spectrum of proteins expressed within the cell which is programmed during differentiation. Thus, an endometrial, oesophageal, or breast cell will respond differently to the hormonal message of hyperinsulinaemia than will a liver or skeletal muscle cell; a mitogenic or cell survival signal will be transduced instead of glucose uptake and glycogenesis.

Increased knowledge about the underlying reasons for the increased risk associated with obesity, the metabolic syndrome, and diabetes should alert clinicians to the possibility that cancers that arise in obese or diabetic individuals are more likely to be driven by the aforementioned. As drugs improve and knowledge about effective biomarkers increases, appropriate and effective stratification of patients will become a reality. Surgeons and oncologists should be made aware of the likely pathways that drive the progression of cancers that arise in the obese, and in those with diabetes mellitus type 2, to ensure that they request appropriate tests and prescribe appropriate therapies.

## Figures and Tables

**Table 1:** Threshold levels to define metabolic syndrome [100].

Characteristic	Threshold level
Abdominal obesity	
Men	>102 cm (40 inches)^$^
Women	>88 cm (35 inches)
Serum HDL	
Men	>40 mg/dL
Women	>50 mg/dL
Triglycerides	≥150 mg/dL
Blood pressure	≥130/85 mm Hg
Fasting glucose	≥110 mg/dL

^$^Abdominal obesity is given as waist circumference.

**Table 2:** Effects of obesity and diabetes mellitus type 2 on risk of different types of cancer.

Type of cancer	Obesity		Diabetes mellitus type 2
	Men	Women	Men and women
Bladder	None	None	1.37 (1.04–1.8) [301]
Breast	—	—	1.2 (1.11–1.3) [302]
Premenopausal	—	0.92 (0.88–0.97)	Not available
Postmenopausal	—	1.12 (1.08–1.16)	Not available
Colorectal	—	—	1.38 (1.26–1.51) [303]
Colon	1.24 (1.20–1.28)	1.09 (1.05–1.13)	Not available
Rectum	1.09 (1.06–1.12)	None	Not available
Endometrium	—	1.59 (1.50–1.68)	2.22 (1.80–2.74) [304]
Gall bladder/biliary tract	None	1.59 (1.02–2.47)	1.43 (1.18–1.72) [305]
Liver	None	None	2.50 (1.80–3.50) [306]
Leukaemia	1.08 (1.02–1.14)	1.17 (1.04–1.32)	Not available
Malignant melanoma	1.17 (1.05–1.30)	None	Not available
Multiple myeloma	1.11 (1.05–1.18)	1.11 (1.07–1.15)	Not available
Non-Hodgkin's lymphoma	1.06 (1.03–1.09)	1.07 (1.00–1.14)	1.79 (1.3–2.47) [307]
Oesophageal adenocarcinoma	1.52 (1.33–1.74)	1.51 (1.31–1.74)	1.3 (1.12–1.50) [25]
Pancreas	None	1.12 (1.02–1.22)	1.94 (1.5–2.4) [308]
Renal	1.24 (1.15–1.34)	1.34 (1.25–1.43)	1.42 (1.06–1.91) [309]
Thyroid	1.33 (1.04–1.70)	1.14 (1.06–1.23)	Not available

The increased relative risks and the 95% confidence limits, in brackets, are given. For obesity, the relative risk ratio is per 5 kg/m^2^ increase in weight [19].

**Table 3:** Affinities of insulin, IGF-1, and IGF-2 for homodimers and heterodimers of isoform A (IR-A) and isoform B (IR-B) of the insulin receptor and type I IGF receptor (IGF-IR).

Ligand	IR-B : IR-B	IR-A : IR-A	IR-A : IR-B	IGF-IR : IGF-IR	IGF-IR : IR-B	IGF-IR : IR-A
Insulin	0.5 nM^$^	0.25 nM	0.5 nM	100 nM	80 nM	70 nM*
IGF-1	100 nM	10 nM	40 nM	0.2 nM	0.3 nM	0.4 nM
IGF-2	10 nM	2 nM	10 nM	0.5 nM	0.5 nM	0.7 nM

^$^Affinities are taken from or are estimated from the different relative affinities reported in [92, 100–102, 104, 310]. *The affinities reported for the hybrid between type I IGF receptor and isoform A of the insulin receptor are discrepant.
